# Conservation benefits of a large marine protected area network that spans multiple ecosystems

**DOI:** 10.1111/cobi.14435

**Published:** 2025-01-09

**Authors:** Joshua G. Smith, Cori Lopazanski, Christopher M. Free, Julien Brun, Clarissa Anderson, Mark H. Carr, Joachim Claudet, Jenifer E. Dugan, Jacob G. Eurich, Tessa B. Francis, David A. Gill, Scott L. Hamilton, Kristin Kaschner, David Mouillot, Peter T. Raimondi, Richard M. Starr, Shelby L. Ziegler, Daniel Malone, Michelle L. Marraffini, Avrey Parsons‐Field, Barbara Spiecker, Mallarie Yeager, Kerry J. Nickols, Jennifer E. Caselle

**Affiliations:** ^1^ National Center for Ecological Analysis and Synthesis University of California, Santa Barbara Santa Barbara California USA; ^2^ Conservation and Science Division Monterey Bay Aquarium Monterey California USA; ^3^ Bren School of Environmental Science and Management University of California, Santa Barbara Santa Barbara California USA; ^4^ Marine Science Institute University of California, Santa Barbara Santa Barbara California USA; ^5^ Research Data Services, Library University of California Santa Barbara Santa Barbara California USA; ^6^ Scripps Institution of Oceanography/Southern California Coastal Ocean Observing System University of California, San Diego La Jolla California USA; ^7^ Department of Ecology and Evolutionary Biology University of California Santa Cruz Santa Cruz California USA; ^8^ National Center for Scientific Research PSL Université Paris, CRIOBE, CNRS‐EPHE‐UPVD Maison de l'Océan Paris France; ^9^ Environmental Defense Fund Santa Barbara California USA; ^10^ Puget Sound Institute University of Washington Tacoma Washington USA; ^11^ Duke Marine Laboratory Nicholas School of the Environment Duke University Beaufort North Carolina USA; ^12^ Moss Landing Marine Laboratories San Jose State University Moss Landing California USA; ^13^ Department of Biometry and Environmental Systems Analysis Albert‐Ludwigs‐University of Freiburg Freiburg Germany; ^14^ MARBEC University of Montpellier, CNRS, IFREMER, IRD Montpellier France; ^15^ Institut Universitaire de France, IUF Paris France; ^16^ Department of Biological Sciences University of New Hampshire Durham New Hampshire USA; ^17^ Habitat Conservation Division, Alaska Regional Office, National Marine Fisheries Service National Oceanic and Atmospheric Administration Juneau Alaska USA; ^18^ Department of Biology California State University Northridge Northridge California USA

**Keywords:** California, fisheries, kelp forests, marine reserve, ocean biodiversity, rocky reefs, surf zone, arrecifes rocosos, biodiversidad oceánica, bosques de algas, California, pesquerías, reserva marina, zona de oleaje, 加利福尼亚, 海洋保护区, 海藻林, 岩礁, 冲浪区, 海洋生物多样性, 渔业

## Abstract

Marine protected areas (MPAs) are widely implemented tools for long‐term ocean conservation and resource management. Assessments of MPA performance have largely focused on specific ecosystems individually and have rarely evaluated performance across multiple ecosystems either in an individual MPA or across an MPA network. We evaluated the conservation performance of 59 MPAs in California's large MPA network, which encompasses 4 primary ecosystems (surf zone, kelp forest, shallow reef, deep reef) and 4 bioregions, and identified MPA attributes that best explain performance. Using a meta‐analytic framework, we evaluated the ability of MPAs to conserve fish biomass, richness, and diversity. At the scale of the network and for 3 of 4 regions, the biomass of species targeted by fishing was positively associated with the level of regulatory protection and was greater inside no‐take MPAs, whereas species not targeted by fishing had similar biomass in MPAs and areas open to fishing. In contrast, species richness and diversity were not as strongly enhanced by MPA protection. The key features of conservation effectiveness included MPA age, preimplementation fisheries pressure, and habitat diversity. Important drivers of MPA effectiveness for single MPAs were consistent across MPAs in the network, spanning regions and ecosystems. With international targets aimed at protecting 30% of the world's oceans by 2030, MPA design and assessment frameworks should consider conservation performance at multiple ecologically relevant scales, from individual MPAs to MPA networks.

## Abstract (es)

Beneficios de la conservación para una extensa red de áreas marinas protegidas que abarca varios ecosistemas

**Resumen:**

Las áreas marinas protegidas (AMP) son herramientas de uso amplio para la conservación a largo plazo de los océanos y la gestión de recursos. Las evaluaciones del rendimiento de las AMP se han centrado casi siempre en ecosistemas específicos de forma individual y rara vez han evaluado el rendimiento a través de varios ecosistemas, ya sea en una AMP individual o a través de una red de AMP. Evaluamos el rendimiento de la conservación de 59 AMP en la gran red de AMP de California, que abarca cuatro ecosistemas primarios (zona de oleaje, bosque de algas, arrecife poco profundo, arrecife profundo) y cuatro biorregiones. También identificamos los atributos de las AMP que mejor explican el rendimiento. Utilizamos un marco meta‐analítico para evaluar la capacidad de las AMP para conservar la biomasa, riqueza y diversidad de peces. A escala de la red y para tres de las cuatro regiones, la biomasa de las especies de interés para la pesca se asoció positivamente con el nivel de protección reglamentaria y fue mayor dentro de las AMP de veda, mientras que las especies que no son de interés para la pesca presentaron una biomasa similar en las AMP y en las zonas abiertas a la pesca. Al contrario, la riqueza y diversidad de especies no mejoraron mucho con la protección de las AMP. Las características clave de la eficacia de la conservación incluyeron la antigüedad de las AMP, la presión pesquera previa a la implementación y la diversidad de hábitats. Los principales factores de la eficacia de las AMP fueron los mismos en todas las AMP de la red, en todas las regiones y en todos los ecosistemas. Con los objetivos internacionales de proteger el 30% de los océanos del mundo para 2030, los marcos de diseño y evaluación de las AMP deben tener en cuenta la eficacia de la conservación a múltiples escalas ecológicamente relevantes, desde las AMP individuales hasta las redes de AMP.

## 跨越多个生态系统的大型海洋保护区网络的保护效益

【摘要】 海洋保护区(marine protected area, MPA)是广泛实施的长期海洋保护和资源管理工具。目前, 对MPA保护成效的评估主要集中在单个特定生态系统上, 很少有研究对单个MPA或整个MPA网络中的多个生态系统的保护成效进行评估。本研究评估了加利福尼亚大型MPA网络中59个MPA的保护成效, 其中包括四种主要的生态系统(冲浪区、海藻林、浅礁、深礁)和四个生物区, 研究还确定了最能解释保护成效的MPA特征。我们利用荟萃分析框架, 评估了MPA保护鱼类生物量、丰富度和多样性的能力。在MPA网络尺度及四个生物区中的三个中, 捕捞目标鱼种的生物量都与监管保护水平呈正相关, 且在禁捕MPA中生物量更大, 而非捕捞目标鱼种在MPA和开放捕捞区内的生物量相近。相比之下, 物种丰富度和多样性并没有因为MPA的保护而显著提高。影响保护成效的关键特征包括MPA建立时长、建立前的渔业压力, 以及生境多样性。影响单个MPA有效性的重要因素在跨区域和跨生态系统的网络的所有MPA中都是一致的。随着“到2030年保护全球30%的海洋”的国际目标的提出, MPA设计和评估框架应考虑从单个MPA到MPA网络的多个生态相关尺度的保护成效。**【翻译:胡怡思;审校:聂永刚】**

## INTRODUCTION

Marine protected areas (MPAs) are an area‐based management strategy primarily focused on long‐term ocean biodiversity conservation. There is global interest in protecting 30% of the ocean by 2030 (30×30) (CBD, 2021; Dinerstein et al., 2019). However, only 8% of the world's oceans are presently covered by MPAs (Bingham et al., 2021; Cinner et al., 2020). Although MPAs are increasingly implemented to provide climate mitigation and resilience (Jacquemont et al., 2022; Roberts et al., 2017), or to increase fisheries yields or profits (Di Lorenzo et al., 2020; Gaines et al., 2010), many were originally envisioned primarily as tools to stimulate the recovery of overfished populations while protecting biodiversity and ecosystem functions (Salm & Clark, 1984). Whether MPAs promote climate resilience (Freedman et al., 2020; Johnson et al., 2022; Smith et al., 2023) or fisheries benefits (Ovando et al., 2021; Radici et al., 2023) is still a matter of debate (Arneth et al., 2023). By contrast, the conservation performance of MPAs—their ability to maintain higher biomass of harvested species, biodiversity, and ecosystem functioning relative to fished locations (Hernández‐Andreu et al., 2024)—is widely documented (Claudet et al., 2008; Edgar et al., 2014; Gill et al., 2017; Lester & Halpern, 2008; Lester et al., 2009; Zupan et al., 2018) and remains the central objective of most MPA management plans (Lopazanski et al., 2023).

Globally, many MPAs are implemented as a single spatially discrete unit (Francour et al., 2001; Grorud‐Colvert et al., 2014). However, there is increasing advocacy for coordinated networks of MPAs that effectively protect biodiversity in and across ecosystems at multiple geographic scales (Jefferson et al., 2021; Jones et al., 2020; Sala et al., 2021; Sève et al., 2023; Visalli et al., 2020). Networks typically include multiple MPAs with meta‐populations connected through propagule dispersal or adult movement, and these networks can encompass many types of ecosystems under various forms of regulatory protection (Gleason et al., 2013; Grorud‐Colvert et al., 2014). We define *ecosystem* as encompassing both the biotic and abiotic components of a particular portion of nearshore coastal environments (i.e., sandy surf zones, kelp forests, shallow reefs, deep reefs) (Marine Life Protection Act, 1999a). Many existing MPA networks aim to protect multiple physical habitats (e.g., hard and soft substrata, etc.) across depth strata and ecosystems (e.g., coral reefs, rock reefs, kelp forests, seagrasses, mangroves, etc.), and expansions in global MPA coverage are thought to be “ecologically representative” and to “efficiently and effectively” protect diverse habitats (CBD, 2021).

Studies of MPA performance have largely focused on specific ecosystems individually (e.g., coral reef, mangrove, rocky reef, kelp forest, and open ocean) and have rarely evaluated performance across multiple ecosystems either in an individual MPA or across an MPA network (see fig. 2c in Gill et al., 2017 for a rare exception, though sample size is limited). Single‐ecosystem and site assessments of MPA performance risk mischaracterizing synthetic effects that may span multiple ecosystems and geographic regions. Conservation performance is likely to vary among ecosystems given differences in community composition, history of fisheries and resource exploitation, vulnerability to anthropogenic stressors, level of protection and compliance, and sensitivity to environmental variation and physical disturbance. As such, there is a need to holistically evaluate the performance of regional MPA networks containing diverse ecosystems within a common framework.

The design and management of MPAs requires understanding the features (e.g., age, size, historic fishing intensity, habitat representation) that promote their efficacy, which could vary in relative importance by ecosystem. Many large‐scale syntheses have revealed features associated with MPA conservation performance, but most have focused on a single type of ecosystem (Edgar et al., 2014; Ziegler et al., 2024) or on pooled data across ecosystems (Claudet et al., 2008; Gill et al., 2017; Lester & Halpern, 2008; Zupan et al., 2018). Further, prior meta‐analyses of MPA performance incorporate data from disparate, single MPAs, often geographically separated, rather than from a large, ecologically connected MPA network. As such, synthetic evaluations are needed to test whether features that confer conservation benefits at the individual ecosystem or MPA scale are also key determinants at the MPA network level (Grorud‐Colvert et al., 2014).

California's (USA) large MPA network presents a unique opportunity to elucidate the impacts of MPAs across diverse fish assemblages inhabiting a variety of ecosystems across multiple habitats and coastal geographies and to identify the MPA features that determine conservation performance. The network contains 124 MPAs that protect 16% of state waters along 1350 km of coastline, spanning approximately 10 degrees of latitude. It was scientifically designed with size and spacing guidelines to promote ecological connectivity (i.e., the dispersal and delivery of propagules; hereafter settlement magnitude) and network functionality (Botsford et al., 2014). Among other goals, the network was explicitly designed to protect “representative and unique marine life habitats in California waters for their intrinsic values” (Marine Life Protection Act, 1999b). It encompasses hard‐ and soft‐bottom habitats, from sandy beaches and the rocky intertidal to depths of 1000 m. For most locations, coordinated long‐term monitoring was initiated in 2007 (the year in which the network expansion began) for the kelp forests, shallow reefs, and deep reefs on the continental shelf. This provides a long and rich time series, in some cases predating MPA establishment, for evaluating the impact of different levels of regulatory protection (e.g., no take, partial take) and MPA features on fish biomass and biodiversity across multiple habitats and ecosystems.

We drew on multiple years of long‐term monitoring data inside and outside 59 MPAs distributed throughout California's large MPA network to examine the impact of regulatory protection on fish biomass, species richness, and biodiversity across surf zone, kelp forest, shallow reef, and deep reef ecosystems. Specifically, we tested the following hypotheses: regulatory protection that limits or prohibits fishing confers positive conservation benefits (fish biomass, richness, and diversity) that vary by protection level (no take vs. partial take) across ecosystems; benefits conferred by regulatory protection are strongest in MPAs that were intensively harvested prior to implementation and for species that are targeted by fisheries; a network of MPAs confers conservation benefits that accrue across ecosystems; and relative outcomes of regulatory protection on conservation performance are explained by MPA features, such as age, size, local preimplementation fishing pressure, larval settlement magnitude, habitat richness, and habitat diversity. We evaluated these features as correlates of MPA conservation performance to inform regulations that could be leveraged when implementing, assessing, or adaptively managing MPA networks around the world (CBD, 2021; Gubernatorial Executive Order N‐82‐20, 2020; Presidential Executive Order 14008: Tackling the Climate Crisis at Home and Abroad).

## METHODS

### Study area and long‐term monitoring

**FIGURE 1:**
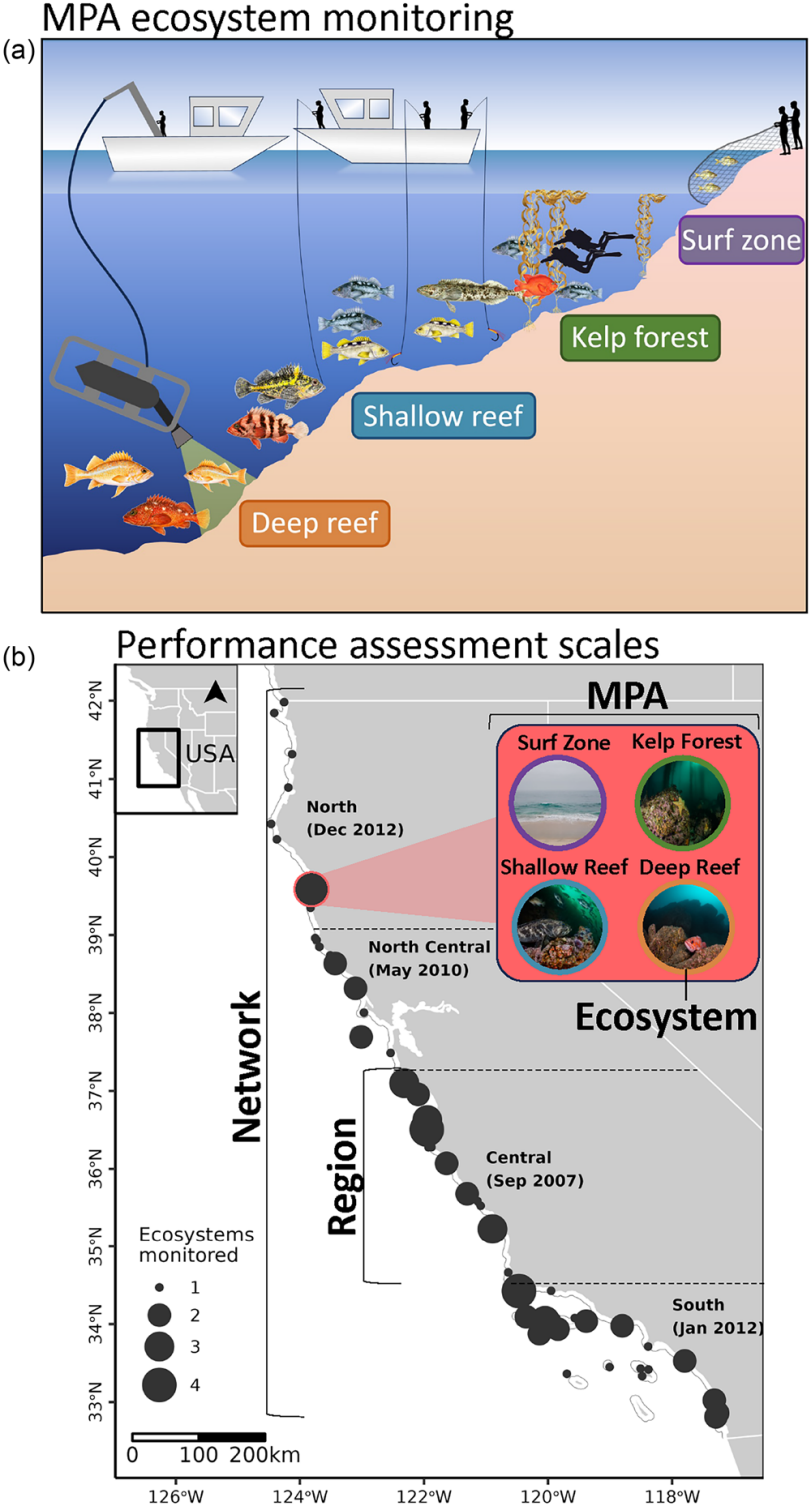
In California's network of marine protected areas (MPAs), (a) general fish sampling methods for the surf zone (seine), kelp forest (scuba), shallow reef (hook and line), and deep reef (remotely operated vehicle) ecosystems and (b) MPAs sampled (*n =* 59) at 4 scales (network [all ecosystems and MPAs in the network], regional [MPAs and ecosystem in a region], ecosystem [1 ecosystem in an MPA], and individual MPA) (MPAs, black circles; circle size, proportional to the number of ecosystems sampled in a given MPA). Fish illustrations provided by A. Caudle at Monterey Bay Aquarium, kelp illustrations provided by J. Kendall‐Bar, and ecosystem images provided by A. Phillips and P. Webster.

California's MPA network consists of 124 MPAs that vary in protection level, including 49 no‐take state marine reserves (SMRs), 10 no‐take state marine conservation areas (SMCAs), 60 partial‐take SMCAs that allow take of specific organisms (with different regulations for each SMCA), and 5 partial‐take state marine recreational management areas (SMRMA) that allow the take of waterfowl (Gleason et al., 2013). All protection levels are hereafter referred to as *MPAs*. California's MPAs were implemented across 4 regions (north, north central, central, and south) at different times from 2007 to 2012, although the network contains some older preexisting MPAs (Van Diggelen et al., 2022) (Figure 1b). For our analyses, we considered 2 types of regulatory protection: no‐take and partial‐take MPAs. An MPA was designated as a de facto no‐take MPA for a particular ecosystem if any allowed partial take was unlikely to directly or indirectly affect the species that reside in that particular ecosystem (e.g., take of salmon in an MPA is unlikely to affect any of our 4 focal ecosystems) (see Appendix S7 & Smith et al., 2023). MPAs with formal and de facto no‐take status were viewed and treated as experiencing equivalent levels of protection throughout the analysis.

Several ecosystem‐specific research groups conduct annual monitoring of California's MPA network. We focused our analyses on 4 ecosystems that have extensive spatial and temporal monitoring coverage (Appendix S15) of fishes across the MPA network: surf zone (sampled using seine nets), kelp forest (depths <20 m, sampled by scuba divers), shallow reef (depths <40 m, but outside kelp, sampled using hook and line), and deep reef (depths 30−100 m, sampled by remotely operated vehicles) (Figure 1a). Each monitoring program uses a paired sampling design where surveys are conducted inside a given MPA and at a neighboring reference area where fishing is allowed. In general, these monitoring programs record the identity (to the lowest taxonomic resolution possible) and length of each fish observed in a systematic survey design. The ecosystem‐specific sampling methods are described in Appendix S1. Ultimately, we included monitoring data from 59 MPAs, generally from 2007 to 2020, although coverage varied by MPA and ecosystem (Appendix S15).

### Conservation performance across the MPA network

We evaluated the conservation performance of the MPA network in terms of targeted and nontargeted fish species (Appendix S4) biomass, richness, and diversity across 4 ecosystems and 2 levels of protection (no take vs. partial take). Biomass was estimated for the surf zone, kelp forest, shallow reef, and deep reef ecosystems based on ecosystem‐specific estimates of fish abundance and body size. Fish length was converted to weight with a standardized biomass parameter table for each species following an extensive literature search. We identified the parameters for other missing species by taking the median conversion parameters for that species reported in FishBase (Froese & Pauly, 2023). We then calculated biomass across all targeted and nontargeted fish species at the smallest replicable unit (e.g., seine, transect, or fishing cell inside or outside an MPA) (Appendix S1). Species richness (number of species) and diversity (Shannon diversity index) were calculated at the MPA level for a given year and ecosystem.

We assessed the conservation performance of the MPA network by evaluating the relative distribution and predictors of fish species biomass, richness, and diversity inside and outside MPAs distributed throughout the network. Among the 124 MPAs in the network, 59 were sampled by at least 1 ecosystem monitoring group over the study period. These MPAs each had a single paired reference area where fishing was allowed. We used a log‐response ratio approach to quantify the relative strength of MPA effects between each pair of protected and fished sites (Hamilton et al., 2010; Ziegler et al., 2022). This yielded a unitless scaled metric of MPA performance that permitted us to compare responses of fish assemblages across multiple monitoring groups in different ecosystems, all sampled using different methods and metrics. The log response ratio for MPA *j* in year *i* (*Y_j,i_*) was calculated as:

(1) $$\;Y_{j,i}=\log\frac{{\bar{X}}_{\mathrm{insid}e_{j,i}}}{{\bar{X}}_{\mathrm{outsid}e_{j,i}}},$$

where ${\bar{X}}_{\mathrm{insid}e_{j,i}}$ and ${\bar{X}}_{\mathrm{outsid}e_{j,i}}$ are the mean performance metrics (biomass, diversity, or richness) across replicate units inside or outside an MPA *j*, respectively, in a given year *i*. We used the log of the response ratio to reduce the variance and scale the response around zero, such that a value above zero indicated a positive effect of the MPA on a given conservation performance metric, and a negative value indicated lower MPA performance (i.e., fish biomass, diversity, or richness was greater outside the MPA). To account for sites where zeros occasionally occurred outside the MPA (precluding inclusion of those MPAs due to an undefined log response ratio), we added a small constant calculated as 10% of the mean of all values for a given ecosystem, year, MPA type (no take or partial take), and site type (inside or outside an MPA). We calculated a fractional constant to account for interannual variability because adding a random constant (e.g., 0.01) could inadvertently skew the response distribution in favor of either the inside or outside locations.

### Syntheses and inference framework

We used 2 statistical approaches to assess 3 metrics (biomass, richness, and diversity) of MPA conservation performance. First, for biomass, we compared the log response ratio of total biomass for targeted and nontargeted fish species with a meta‐analytic framework. Before our analyses, we classified the target status for each species with a 2‐stage approach. We first used records of commercial and recreational fisheries catch from 2000 to 2022 (Free et al., 2022) to identify species caught in California's fisheries. We then reclassified low‐volume bycatch species as nontargeted and cryptic species that are targeted by fishers but poorly represented in catch records as targeted (Appendix S4). The null assumption was that nontargeted fish species respond less strongly (either positively or negatively) than targeted species to MPA implementation because they only experience indirect effects of protection on the ecosystem (e.g., increased predation or competition resulting from recovery of targeted species). As a result, they act as a type of control for variation in environmental conditions that may affect all species similarly. We inferred that a stronger positive response of targeted species relative to nontargeted species signifies the predicted effects of MPA protection. Second, for species richness and diversity, we compared the distribution of the log response ratios for each ecosystem and evaluated significance with a 2‐tailed *t* test on the log response ratio. This was the most appropriate form of analysis because richness and diversity were each calculated across replicates in an MPA (i.e., at the level of the MPA rather than haul, transect, or fishing cell) and because sampling was often depth stratified and many species are associated with particular depths. Therefore, it was inappropriate to calculate these 2 performance metrics at a smaller scale (e.g., seine, transect, or fishing cell).

We used a meta‐analytic framework to evaluate the effect of regulatory protection on fish biomass across the evaluated MPAs and ecosystems. This analysis used biomass as the focal performance metric because it contained both an effect size and associated unit variance for each ecosystem and MPA. However, the shallow reef ecosystem used hook‐and‐line sampling, the same gear used to target nearshore recreational fish species, meaning that nontargeted fish species were not sufficiently sampled for inclusion in the meta‐analysis. The biomass effect size for each ecosystem at a given MPA was modeled as the log ratio (Equation 1). When data were collected in an individual MPA over time, we retained only the most recent results to reflect the longest duration of protection for a given ecosystem (Zupan et al., 2018). The within‐study variance of each unique ecosystem‐MPA combination was calculated as:

(2) $$\;v_{E_{j}}=\frac{\sigma_{\mathrm{insid}e_{E_{j}}}^{2}}{n_{\mathrm{insid}e_{E_{j}}}*{\bar{X}}_{\mathrm{insid}e_{E,i}}}+\frac{\sigma_{\mathrm{outsid}e_{E_{j}}}^{2}}{n_{\mathrm{outsid}e_{E_{j}}}*{\bar{X}}_{\mathrm{outsid}e_{E,i}}},$$

where ${\bar{X}}_{\mathrm{insid}e_{E,i}}$ and ${\bar{X}}_{\mathrm{outsid}e_{E,i}}$ are the mean biomass estimates (targeted or nontargeted, separately) for a given ecosystem E (surf zone, kelp forest, shallow reef, deep reef) inside and outside, respectively, of MPA j in the most recent year; σ is the standard deviation associated with each mean at $E_{j}$; and n is the number of replicates (seines, transects, or fishing cells) used to estimate the mean for $E_{j}$.

The conservation performance (*R*) of an individual MPA (*n* = 59), region (*n* = 4), or ecosystem (*n* = 4) (Figure 1b) was calculated as a weighted average of the effect size as a function of target status (targeted or nontargeted) as:

(3) $$\bar{R}=\frac{\sum_{i=1}^{n_{i}}\left(w_{i}Y_{j}\right)}{\sum_{i=1}^{n_{i}}w_{i}},$$

where $w_{i}$ is the inverse of the within $v_{E,i}$ and between ${\hat{\tau }}^{2}$ study variance defined as:

(4) $$w_{i}=\frac{1}{v_{E_{j}}+{\hat{\tau }}^{2}}$$

and

(5) $${\hat{\tau }}^{2}=\frac{Q-\left(k-1\right)}{c},$$

where *k* is the number of ecosystems, *c* is a constant equal to k−1, and Q is the overall heterogeneity given by:

(6) $$Q=\sum_{i=1}^{n_{i}}w_{i}{\left(Y_{j}-\bar{R}\right)}^{2}.$$

### MPA features and conservation performance

To evaluate the network‐level predictors of MPA conservation performance across all ecosystems and sampled MPAs, we constructed a meta‐generalized additive model (meta‐GAM) with the mgcv package in R (Wood, 2011). We evaluated the impact of 8 MPA features on conservation performance (log ratio effect size): MPA age (year), MPA size (square kilometers), habitat diversity (number of habitats and their relative area), habitat richness (number of distinct habitats), proportion of MPA with rocky bottom, local pre‐MPA fisheries landings, ecosystem‐specific estimated larval settlement, and total estimated larval settlement to an MPA. The last 2 features were estimated from ROMS (Regional Ocean Modeling System) larval dispersal and models (Appendix S1). We restricted the analyses to no‐take MPAs and targeted fish species to parse the overall relationship between performance and each predictor variable while holding the most restricted level of protection (no take) constant. To further explore the ecosystem‐level predictors of conservation performance, we used a series of random forest models on individual ecosystems. Details on the random forest models are in Appendix S1, and details on how each MPA feature was defined and derived are in Appendices S5 and S6.

To construct the meta‐GAM, MPA features were added as smoothing terms and year was included as a cyclic cubic regression spline to account for periodic trends over time in the data. The model included all sampled no‐take MPAs and ecosystems weighted using $w_{i}$ (Equation 4). We used a Gaussian link function and cubic spline to determine the optimal level of smoothing for each predictor. Model selection was conducted using generalized cross‐validation (GCV) with a forward selection procedure (Marra & Wood, 2011).

The data that support the findings of this study are openly available in DataONE for the surf zone (Dugan & Marraffini, 2022), kelp forest (Carr et al., 2021), shallow reef (Brooks et al., 2022), and deep reef (Cieri et al., 2022) ecosystems. Additional metadata are provided in Appendix S5. Source code supporting the analyses presented in this study is available at https://github.com/NCEAS/ca‐mpa.

## RESULTS

### Network‐wide performance

**FIGURE 2:**
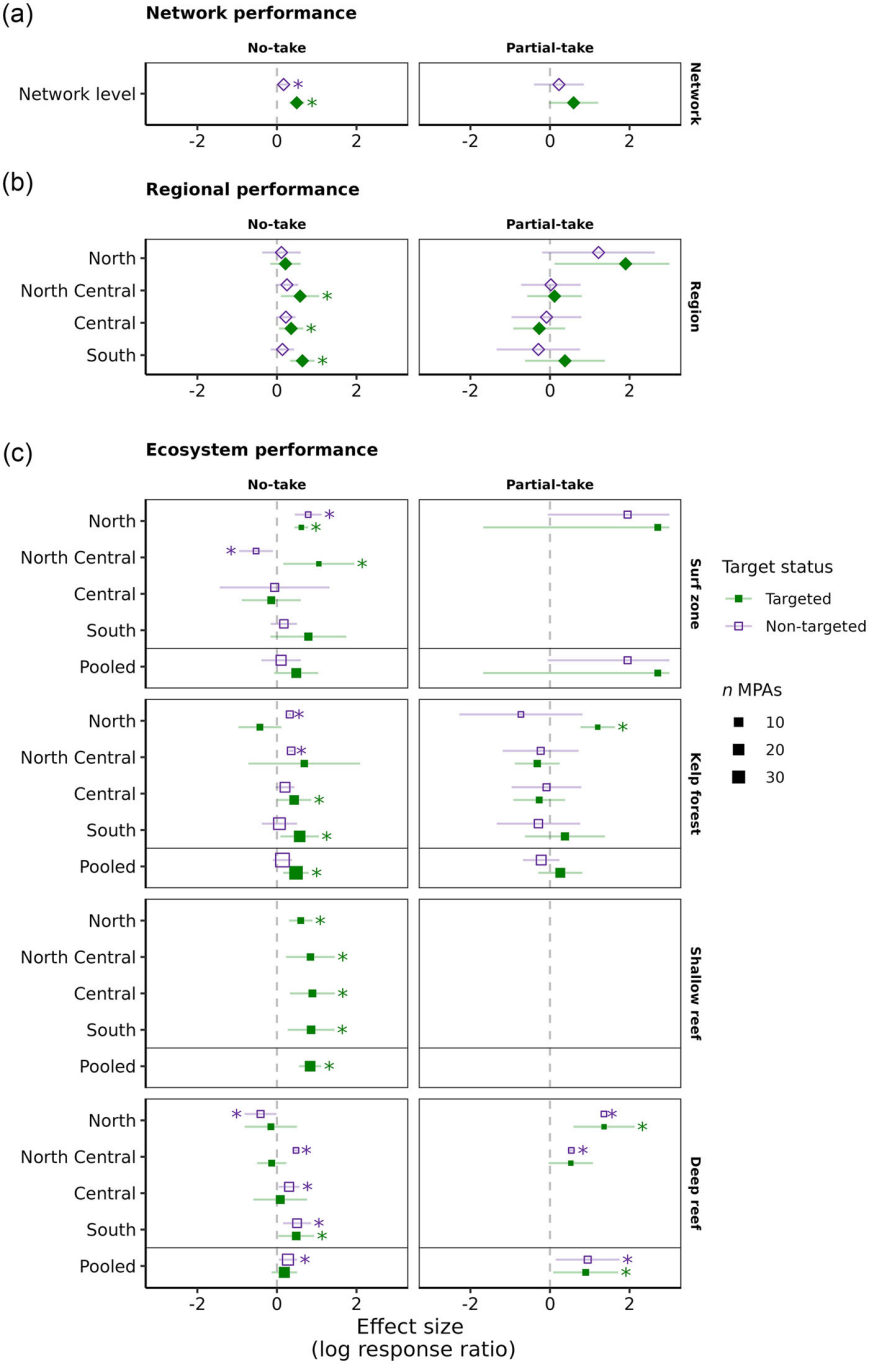
Biomass response ratios in California's network of marine protected areas for targeted (filled green squares and diamonds) and nontargeted (open purple squares and diamonds) fish species by protection level (no take vs. partial take) across: (a) the network and by (b) region and (c) ecosystem (squares or diamonds, mean effect size across MPAs for a given scale [see Figure 1b]; horizontal lines, 95% confidence intervals [upper bounds >3 truncated to ease visualization]; asterisks, *p* < 0.05; square size, relative number of MPAs included in the effect size; diamonds, weighted effect sizes [not scaled by size]; vertical dotted lines, no effect; positive values, higher biomass in MPAs; negative values, higher biomass outside MPAs; pooled, meta‐analytic effect size across all regions for a given ecosystem; data gaps, MPAs or species assemblages not sampled [e.g., nontargeted species in the shallow reef due to hook‐and‐line sampling]).

At the scale of the entire statewide network of MPAs (i.e., results pooled across ecosystems, regions, and MPAs) (Figure 1), targeted (i.e., fished) fish biomass was positively associated with regulatory protection and was significantly greater inside no‐take MPAs compared with areas that allowed take (effect size [E.S.] = 0.497, *p* < 0.001) (Figure 2a & Appendix S9). Nontargeted fish species biomass was greater inside no‐take MPAs (E.S. = 0.167, *p* = 0.049) but was not significantly higher in partial‐take MPAs relative to reference sites (areas that allow fishing). For partial‐take MPAs, fish biomass was not significantly different between targeted and nontargeted species. However, targeted species biomass was higher inside MPAs than in reference sites (Figure 2a). Fish species diversity and richness did not respond to any protection level in any ecosystem (Appendix S16).

### Regional performance

The MPA‐level conservation performance differed geographically by region. Three out of 4 regions exhibited significantly higher targeted fish biomass inside no‐take MPAs when pooled across ecosystems (Figure 2b & Appendix S10). These 3 regions (north central coast, central coast, south coast) also had slightly higher nontargeted fish biomass in no‐take MPAs, although this result was not significant (Appendix S10). The south coast, the region with the largest human population size and fishing pressure, showed the strongest overall positive effect of regulatory protection in no‐take MPAs for targeted species (E.S. = 0.641, *p* < 0.001) (Appendix S10). The only regionally significant effect size for partial‐take MPAs was for targeted species in the north coast (E.S. = 1.901, *p* = 0.037) (Appendix S10). The other 3 regions (north central, central, south) had similar effect sizes for targeted and nontargeted species in partial‐take MPAs.

### Ecosystem‐specific performance

In the surf zone, MPA conservation performance was generally positive but varied by region and MPA type with no regional gradient (Figure 2c, Appendices S11 & S12). Across all MPAs for the surf zone ecosystem, the pooled response ratios were higher inside no‐take and partial‐take MPAs, but this result was nonsignificant (Appendix S11). The north coast exhibited strongly positive and significant response ratios for both targeted (E.S. = 0.613, *p* < 0.001) and nontargeted (E.S. = 0.785, *p* < 0.001) species for the single no‐take MPA surveyed (Appendix S12). Targeted fish biomass was also significantly higher inside the no‐take MPA in the north central coast region (E.S. = 1.053, *p* = 0.02), whereas nontargeted species biomass was higher outside (E.S. = −0.525, *p* = 0.015). Both targeted and nontargeted fish biomass for the central coast showed slightly negative, though nonsignificant, effects of no‐take MPAs. For south coast no‐take MPAs, response ratios were higher inside MPAs but this result was not significant.

The kelp forest ecosystem exhibited significantly higher fish biomass inside no‐take MPAs for targeted species when pooled across all regions (E.S. = 0.479, *p* = 0.004) (Figure 2c & Appendix S11). A strong regional gradient in MPA performance was also apparent for kelp forest fishes, with the south coast (E.S. = 0.571, *p* = 0.02) and central coast (E.S. = 0.433, *p* = 0.048) regions showing strong and significant positive effects inside no‐take MPAs, the north central coast exhibiting a positive but nonsignificant effect, and the north coast exhibiting a negative, though nonsignificant, effect (Figure 2c). In partial‐take MPAs, targeted fish biomass was significantly higher in the north coast (E.S. = 1.199, *p* < 0.001) (Appendix S12) but nonsignificant for all other regions and overall.

Among the 4 ecosystems, positive biomass response ratios were most pronounced in the shallow reef ecosystem (Figure 2c & Appendix S11). Targeted fish biomass was significantly higher in no‐take MPAs in all 4 regions (Appendix S12) and when pooled across regions (E.S. = 0.833, *p* < 0.001) (Appendix S11). Because of the gear type (hook and line) used to sample shallow reef fishes, nontargeted species were rarely caught and thus not included. The shallow reef ecosystem also selectively sampled only no‐take MPAs, and, therefore, partial‐take MPAs were not included in the analysis.

Finally, in the deep reef ecosystem, the overall effect size was significantly positive for nontargeted fish biomass in no‐take MPAs when pooled across regions (E.S. = 0.276, *p* = 0.018) (Figure 2c & Appendix S11). However, this result was likely influenced by the south coast region, which showed a very positive and strong effect size for nontargeted fish biomass in no‐take MPAs (E.S. = 0.506, *p* = 0.005) (Appendix S12). Targeted fish biomass was slightly higher in no‐take MPAs when pooled across regions, though this relationship was not significant (Figure 2c & Appendix S11) and varied among regions (Figure 2c). Among the 4 ecosystems, the deep reef had the strongest positive effect size in partial‐take MPAs for both targeted and nontargeted species (Appendix S12).

### MPA‐level performance

**FIGURE 3:**
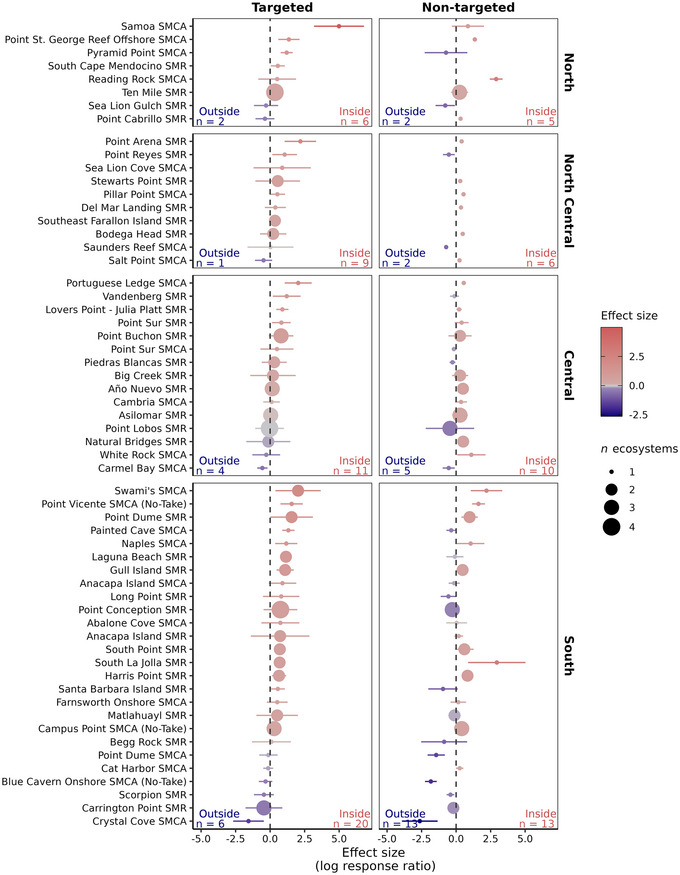
Biomass response ratios in 59 California marine protected areas (MPAs) for targeted and nontargeted fish species for by region (points, weighted [by inverse of variance] mean effect size for a given MPA across all ecosystems; point size, number of ecosystems included in the effect size; MPA order, descending effect size in each region; SMRs, no‐take state marine reserves; SMCAs, partial‐take state marine conservation areas [see Appendix S7 for SMCAs classified as de facto MPAs for some of their constituent ecosystems]; error bars, 95% confidence intervals; vertical dashed line, null assumption of comparable biomass estimates inside and outside MPAs; positive values, greater biomass inside MPAs; negative values, greater biomass outside MPAs; *n*, MPA sample size).

Across the network of 59 sampled MPAs, the effect of regulatory protection was positive for the majority of MPAs when pooled across ecosystems (Figure 3). Targeted fish species biomass was significantly higher in 22 out of 59 MPAs (37% of MPAs), although 46 (78% of MPAs) showed positive effect sizes for targeted fish biomass. Nontargeted fish species biomass was also elevated inside MPAs (Figure 3 & Appendix S13). Twenty‐two out of 56 MPAs (37%) had significantly higher biomass in the MPAs, and 34 had elevated (57%, though nonsignificant) biomass. However, there were proportional differences between targeted and nontargeted species in individual regions. In the north coast, 6 out of 8 (75%) MPAs had higher biomass for targeted species, whereas nontargeted species had greater biomass in 5 out of 7 MPAs (71%). In the north central region, 9 out of 10 MPAs had higher targeted biomass (90%), and 6 out of 8 (75%) MPAs had higher nontargeted biomass. In the central coast region, 11 of 15 MPAs (73%) had higher targeted species biomass, whereas 10 out of 15 (66%) had higher nontargeted biomass. Finally, in the south coast region, 20 out of 26 MPAs (77%) had higher targeted biomass, whereas the number of MPAs with higher or lower nontargeted biomass was equally distributed (13 out of 26, 50%).

### Network‐wide predictors of conservation performance

**FIGURE 4:**
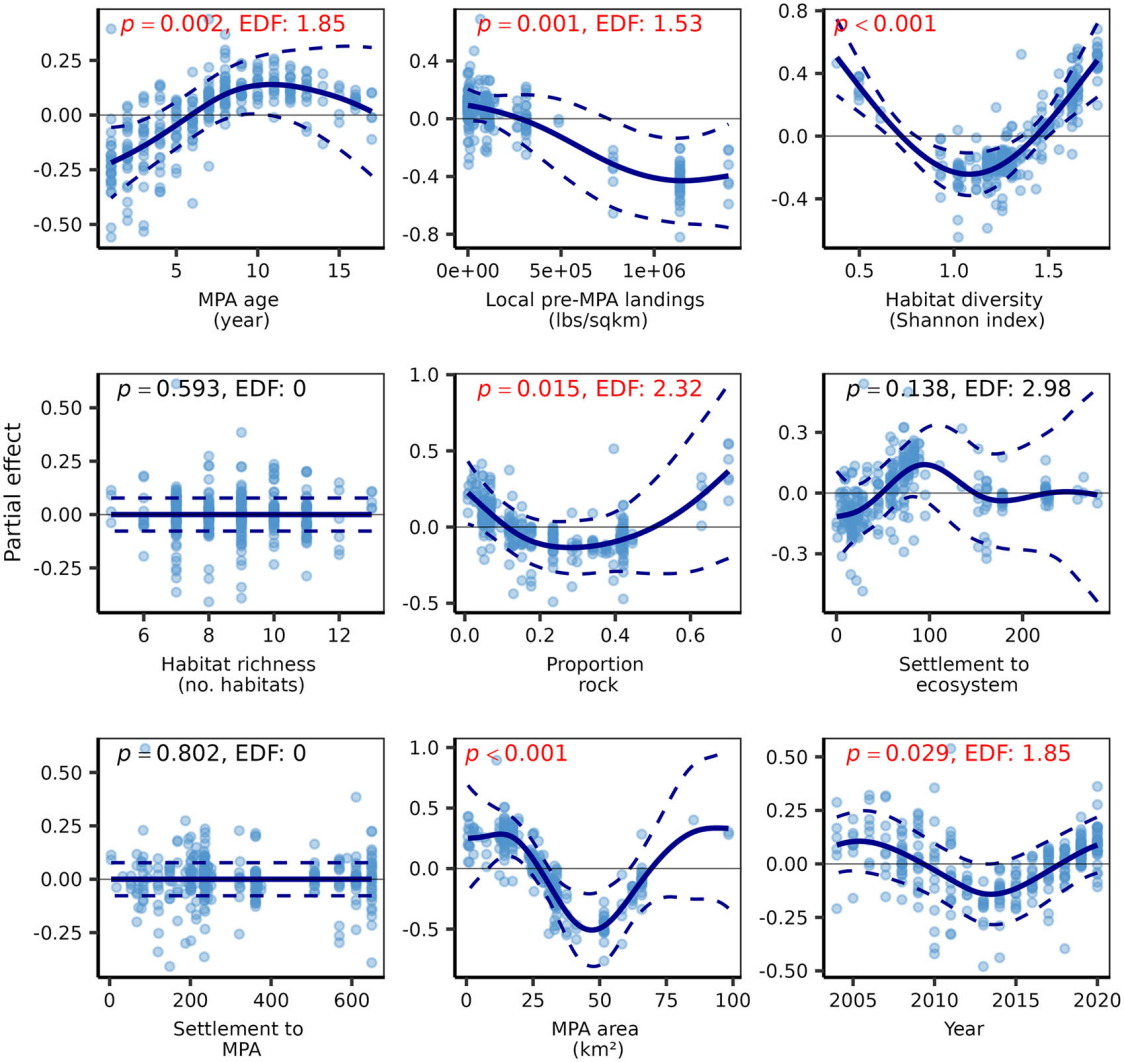
Partial effects of California marine protected area (MPA) features on conservation performance from a meta‐generalized additive model (solid blue lines, shape of the relationship between each MPA feature and performance [response ratio effect size]; dashed lines, 95% confidence intervals; blue points, residuals; EDF, effective degrees of freedom; red, statistically significant relationships). See Appendices S5 and S6 for details on how each feature was defined and derived.

The meta‐generalized additive model captured a moderate amount of variation in the data (GCV = 0.073, *n* = 292, adjusted *r* ^2^ = 0.153, *p* < 0.001) and revealed highly influential MPA features that explained performance (response ratio effect size) across ecosystems (Figure 4). Results of the model indicated that MPA age (*p* = 0.002, EDF = 1.85), local preimplementation landings (*p* = 0.001, EDF = 1.53), habitat diversity (*p* < 0.001, EDF = 1), and proportion of rocky substratum (*p* < 0.05, EDF = 2.32) were the strongest significant correlates of conservation performance (Appendix S14). Conservation performance (i.e., the difference in fish biomass between MPA and reference sites) significantly increased with increasing MPA age and habitat diversity. However, before‐implementation fisheries landings were slightly inversely related to performance (*p* = 0.001, EDF = 1.57). The impacts of proportion rock and MPA size, while statistically significant, were highly nonlinear, but larger MPAs and those with a greater proportion of rock generally had stronger positive responses, especially at the upper value range (Figure 4). Larval settlement was not significantly correlated to conservation performance.

## DISCUSSION

We explored the performance of MPAs across a scientifically designed network and found that conservation benefits accrue across a mosaic of surf zone, kelp forest, shallow reef, and deep reef ecosystems. Although many studies have evaluated MPA performance in individual protected areas or across single ecosystems, few have explored the conservation outcomes of networks of MPAs across multiple ecosystems (but see Goetze et al., 2021). Our findings highlight opportunities for strategic planning and assessment frameworks that maximize conservation impact.

Our analyses indicated that among all MPA features, MPA age and habitat diversity were the strongest overall predictors of performance, where older MPAs with greater habitat diversity generally had more fish biomass than their associated reference sites. Size of an MPA, which has previously been shown to drive MPA effectiveness (Claudet et al., 2008), was less important and highly nonlinear in our system, perhaps due to the size and spacing guidelines for network design that resulted in relatively small variation in MPA area across the network. The California network was designed with specific attention to ensuring habitat representation and replication in MPAs and in each region (Gleason et al., 2013) based on increasingly sophisticated spatial mapping tools throughout the design process. Incorporating a diversity of habitats in individual MPAs and across networks not only increases the magnitude of biodiversity protection, but also provides resilience to disturbances including future climate impacts (Wilson et al., 2020). However, despite widespread discussion and theoretical treatment of this MPA trait, to our knowledge, ours is the first quantitative test of the importance of habitat diversity to MPA performance.

Another critical and widely discussed design principle for MPA networks is connectivity (Goetze et al., 2021; Sève et al., 2023). In an effective network, organisms must be able to travel or disperse through larval connectivity among protected areas. Indeed, the California network design phase incorporated perhaps one of the most detailed sets of MPA size and spacing guidelines to date, taking into account generalized larval dispersal distances and patterns of ocean circulation (Gleason et al., 2013). By using different size and spacing of protected areas, a network can protect species with different life histories and behavioral characteristics and may offer better conservation performance than single large protected areas (McLeod et al., 2009; Moffitt et al., 2011). Yet, here, using realistic, estimated settlement magnitude from larval dispersal modeling as a proxy for connectivity, we did not find a significant effect when synthesized across ecosystems. However, estimated settlement magnitude was important on an individual ecosystem level. These 2 contrasting results likely reflect that organismal‐level estimates of larval durations are needed to accurately assess the relative importance of connectivity for individual ecosystems to entire MPAs.

Our finding of higher targeted fish species biomass inside no‐take MPAs is likely the result of regulatory protection (i.e., an emergent effect) rather than a placement effect (i.e., placed in an area with high initial biomass or habitat quality) because MPA age was the strongest determinant of biomass response ratios in the majority of ecosystems studied. Emergent effects are expected to increase in magnitude inside the MPA relative to the outside reference location over time as a result of regulatory protection (i.e., continued fishing outside the MPA restricts increases in biomass), until spillover replenishes neighboring unprotected areas (Di Lorenzo et al., 2020; Goñi et al., 2008, 2010). However, placement effects occur when an MPA is implemented in an area with higher preexisting biomass (or more suitable habitat) than the reference area (Claudet & Guidetti, 2010; Gaines et al., 2010; Roberts, 2000). If observed higher biomass inside MPAs was the result of a placement effect, MPA age would not be a strong determinant of performance, as high preexisting biomass would remain stable through time. However, placement effects may provide other positive enabling conditions (such as more suitable habitat) that may be important in the design phase of networks. Furthermore, our finding of a positive (but lower relative to targeted species) increase in the biomass of nontargeted fish species supports an MPA effect because nontargeted species should not have a direct response to protection and, therefore, serve as a type of control measure (Caselle et al., 2015; Claudet et al., 2010; Ovando et al., 2021).

Regional differences in MPA performance may be the result of a combination of sampling limitations, variation in species life history traits, or environmental perturbations (Marraffini et al., 2024; Ziegler et al., 2024). Disentangling these effects becomes even more challenging when evaluating large MPA networks that span biogeographic regions. For example, ecosystems in the north coast region were comparatively less sampled than the south coast, potentially limiting the power to detect an MPA effect. The MPAs along the north coast are also the youngest in the network, further limiting the effect size for these MPAs given our finding that effect sizes increase with age. However, the north coast is also characteristically dominated by rockfish (*Sebastes* spp.) species that are long‐lived, late to mature, and have more episodic year‐class recruitment success (Love et al., 2002), which could contribute to a slower MPA response in this region. Additionally, in 2014−2016, a large Pacific marine heatwave affected the north, north central, and central coast regions (McPherson et al., 2021). This marine heatwave occurred only 2 years after full implementation of the MPA network (Smith et al., 2023) and was followed by El Niño events in 2018 and 2023 (Leising et al., 2024). Environmental perturbations, such as marine heatwaves and El Niños, and long‐term environmental change, can reduce the ability to detect MPA effects (Hopf & White, 2023), especially in locations where MPAs were not originally designed to provide climate resilience (Arafeh‐Dalmau et al., 2023; Smith et al., 2023). In our study system, the impacts of the marine heatwave event on fish biomass remain unclear, but trends in biomass and biodiversity over time inside and outside MPAs were likely affected by this environmental perturbation (Free et al., 2023; Freedman et al., 2020; Ziegler et al., 2023).

Across the 4 ecosystems included in our analyses, we hypothesized that MPA responses are strongest in MPAs where preimplementation fishing was greatest. Although historic fishing intensity explained a moderate amount of variation in biomass across the MPA network, observed differences in performance between ecosystems could be the result of sampling gear types or other regulatory protection measures. For example, our analyses showed that conservation performance was strongest for shallow reef fishes. The shallow reef monitoring group used hook and line sampling, which disproportionately selects older, larger individuals and may reflect a higher sampling of size ranges that are typically targeted by fisheries. Conversely, visual sampling conducted in the kelp forest and deep reef ecosystems as well as surf zone sampling with beach seines are nonselective and result in high proportions of smaller individuals, which do not receive the same fishing pressure as larger individuals of the same species (Marraffini et al., 2024). The deep reef ecosystem showed comparatively lower responses in targeted fish biomass relative to the other ecosystems. Many of the locations sampled in the deep reef ecosystem were in rockfish conservation areas, which have, since 2002, restricted fishing across large swaths of the West Coast of the United States to depths <36 to 100 m to reduce the incidental catch of overfished species (Mason et al., 2012). These depth closures likely created additional protection for fishes outside the state's network of MPAs (Keller et al., 2022), which could explain the less pronounced difference in the effect size for the deep reef ecosystem.

Our finding of no differences in taxonomic diversity and richness inside and outside MPAs is consistent with other studies that explored these metrics of MPA performance (Blowes et al., 2020; Ramírez‐Ortiz et al., 2020). The primary regulation associated with the California MPA network, and many global MPAs, involves a restriction or reduction of fishing activities, which generally affects fish assemblages through the total number of individuals, size structure, and their relative abundance (proportional representation of each species). Therefore, the fishes most affected by fishing preimplementation are likely to see the greatest biomass response (Caselle et al., 2015; Claudet et al., 2010). However, because diversity includes the number of species and their evenness, the taxonomic diversity of fishes may not change as a result of regulatory implementation or there may be more nuanced increases in evenness without changes in the absolute number (richness) of species. Other taxonomic diversity indices, such as functional or trait‐based evaluations, could provide additional pathways to evaluate MPA performance (Dalongeville et al., 2022; Dee et al., 2016; Rincón‐Díaz et al., 2018; Ziegler et al., 2023). This effect should be considered when proposing new MPAs or networks with goals of increasing biodiversity, especially in locations with other ecosystem management tools in place (e.g., water quality, traditional fisheries management, tribal or indigenous management).

Ultimately, our findings for this MPA network suggest that positive conservation benefits of MPAs can accrue across multiple ecosystems. We found that MPA features, such as age, habitat diversity, and local preimplementation landings, are highly influential on conservation performance. Although the conservation performance of MPAs can vary across individual MPAs, coastal geographies, and ecosystems, a network can provide net positive benefits. With international targets aimed at protecting more of the world's oceans (including 30 by 30) (CBD, 2021; Dinerstein et al., 2019), MPA design and assessment frameworks should consider performance at multiple ecologically relevant scales, spanning individual MPAs to multiple ecosystems and networks.

## Supporting information



Appendices S1−S17
