# Supplementary material for: Conservation benefits of a large marine protected area network that spans multiple ecosystems
Source: Conserv Biol. 2025 Jan 9;39(4):e14435. doi: 10.1111/cobi.14435 (PMC12309648; doi:10.1111/cobi.14435)
Supplement: Supplementary file 1 — Appendices S1−S17 [file COBI-39-e14435-s001.docx]

# **Conservation benefits of a large marine protected area network that spans multiple ecosystems**

**Appendices S1-S17**

Joshua G. Smith^1,2^*, Cori Lopazanski^3^, Christopher M. Free^3,4^, Julien Brun^20^, Clarissa Anderson^5^, Mark H. Carr^6^, Joachim Claudet^7^, Jenifer E. Dugan^4^, Jacob G. Eurich^8,1^, Tessa B. Francis^9^, David A. Gill^10^, Scott L. Hamilton^11^, Kristin Kaschner^12^, David Mouillot^13,14^, Peter T. Raimondi^6^, Richard M. Starr^11^, Shelby L. Ziegler^11,15^, Daniel Malone^6^, Michelle L. Marraffini^4^, Avrey Parsons-Field^4^, Barbara Spiecker^4,16^, Mallarie Yeager^6,17^, Kerry J. Nickols^18,19^, Jennifer E. Caselle^4^

^1^ National Center for Ecological Analysis and Synthesis, University of California, Santa Barbara, Santa Barbara, CA, USA

^2^ Conservation and Science Division, Monterey Bay Aquarium, CA, USA

^3^ Bren School of Environmental Science and Management, University of California, Santa Barbara, Santa Barbara, CA, USA
^4^ Marine Science Institute, University of California, Santa Barbara, Santa Barbara, CA, USA

^5^ Scripps Institution of Oceanography/Southern California Coastal Ocean Observing System, University of California, San Diego, La Jolla, California, USA

^6^ Department of Ecology and Evolutionary Biology, University of California Santa Cruz, Santa Cruz, CA, USA

^7^ National Center for Scientific Research, PSL Université Paris, CRIOBE, CNRS-EPHE-UPVD, Maison de l’Océan, Paris, France

^8^ Environmental Defense Fund, Santa Barbara, CA, USA

^9^ Puget Sound Institute, University of Washington, Tacoma, Washington, USA

^10^ Duke Marine Laboratory, Nicholas School of the Environment, Duke University, Beaufort, North Carolina, USA

^11^ Moss Landing Marine Laboratories, San Jose State University, Moss Landing, CA, USA

^12^ Department of Biometry and Environmental Systems Analysis, Albert-Ludwigs-University of Freiburg, Freiburg, Germany

^13^ MARBEC, University of Montpellier, CNRS, IFREMER, IRD, Montpellier, France

^14^ Institut Universitaire de France, IUF, Paris, France

^15^ Current affiliation: Department of Biology, Villanova University, Villanova, PA, USA

^16^ Department of Biological Sciences, University of New Hampshire, Durham, NH, USA

^17^ Habitat Conservation Division, Alaska Regional Office, National Marine Fisheries Service, National Oceanic and Atmospheric Administration, Juneau, AK, USA

^18^ Department of Biology, California State University Northridge, Northridge, CA, USA
^19^ Current affiliation: Ocean Visions, Leesburg, VA, USA

^20^ Research Data Services, Library, University of California Santa Barbara, Santa Barbara, CA , USA

*** Corresponding author:** National Center for Ecological Analysis and Synthesis, University of California, Santa Barbara, Santa Barbara, CA, USA; [josgraysmith@gmail.com](mailto:josgraysmith@gmail.com)

## **Appendix S1**

#### *Monitoring sampling design*

**Surf zone:** Surf zone monitoring began in 2019 and has been conducted in 13 unique MPAs paired with reference sites (**Appendix S15**). Surveys of surf zone fish at each MPA and reference site were conducted three times a year (between June - November) during standardized tide windows (less than equal to 1 m) and safe ocean conditions. On each survey date, six beach seine hauls (net dimensions: 15.25 m long x 1.8 m high, 1 cm mesh with two poles attached on each end and central a 1.8 m x 1.8 m x 1.8 m bag) were conducted in the shallow surf zone (<1.5 m). Fish from each haul were identified, counted, and measured (standard and/or total length for the first 30 individuals of each species per haul) before being released at the capture site. Total length represents the distance from the tip of the snout to the most posterior part of the caudal fin without compressing the tail. Standard length represents distance from the tip of the snout to the end of the last vertebrae. Conversion tables based on published relationships were used to convert standard lengths to total lengths as necessary. For biomass estimates, we resampled from the size-frequency distribution of the 30 subsampled individuals to estimate the total length-frequency distribution of all individuals for a given haul. Surf zone monitoring is led by the Dugan lab at University of California, Santa Barbara.

**Kelp forest:** Kelp forest monitoring began in 1999 on the South and Central Coasts, 2010 on the North Central Coast, and 2014 in the North Coast, and has been conducted in 46 unique MPAs paired with reference sites MPAs (**Appendix S15**). Replicate sampling sites are distributed within each MPA and at nearby “reference” sites of comparable habitat characteristics to those inside the MPA. Each survey site typically consists of a rectangular area, extending 150 m parallel with the shore. The onshore-offshore dimension varies depending on the width of the reef and the offshore distance of the 20 m isobath (depth). Typically, two to four sites inside and two to four sites outside each MPA were surveyed with the number and shape of sites varying depending on habitat (e.g., onshore-offshore steepness of the reef) and longshore width of the MPA. To characterize the ecological community throughout each sampling site, belt transects (30 m x 2 m) were distributed across a depth gradient from the 5 m to the 20 m isobath at each site. Surveys were generally conducted in 1-2 visits to a site per year from June/July through October/November each year. Diver surveys were conducted to estimate the density and size distribution of all conspicuous fishes along replicate transects at three levels (bottom, midwater, canopy) in the water column. More details for each of the methods are described in [(Caselle et al., 2015; Hamilton et al., 2010; Malone et al., 2022)](https://www.zotero.org/google-docs/?05qDKj). For our analyses, we excluded canopy surveys because of temporal inconsistencies and combined the midwater and benthic surveys to estimate total fish density along replicate transects inside and outside of MPAs. Kelp forest monitoring is led by the Caselle lab at University of California, Santa Barbara.

**Shallow reef:** Within the boundaries of each sampled MPA and reference area, 500 m by 500 m fixed grid cells were delineated in rocky habitats shallower than 40 m depth (to limit fishing mortality associated with barotrauma). Surveys were conducted annually along the Central Coast from 2007–2020 and expanded to the North and South Coasts in 2017 to include a total of 15 unique MPAs paired with reference sites. Volunteer anglers were recruited from various fishing clubs, online fishing websites, and from previous collaborative studies. Before each day of fishing, four grid cells in a given MPA or reference site were randomly chosen for sampling. Captains were instructed to locate three suitable fishing locations within each grid cell to complete fishing drifts with a goal of 15 minutes each. For each drift, information on the number of anglers, time spent fishing (minutes), location (GPS coordinates), depth (ft), habitat relief, and other environmental variables were recorded. Anglers used a standardized set of fishing gear across the entire state (shrimp flies with squid bait) and within regions to capture a variety of species and cover the spectrum of typical hook-and-line fishing gear used by anglers [(Starr et al., 2015; Yochum et al., 2011; Ziegler et al., 2022)](https://www.zotero.org/google-docs/?55bth3). Captured fishes were identified to species, measured to the nearest cm, and released. Lengths reported are total length, defined as the distance from the tip of the snout to the most posterior part of the caudal fin without compressing the tail. Shallow reef monitoring is conducted by the Caselle lab at University of California, Santa Barbara and the Hamilton lab at Moss Landing Marine Laboratories.

**Deep reef:** Deep reef monitoring began in 2005 on the South Coast, 2007 on the Central Coast, 2011 on the North Central Coast, and 2014 in the North Coast, and has been conducted in 19 unique MPA-reference site pairs (**Appendix S15**). Remotely Operated Vehicle (ROV) survey sites were initially identified using bathymetric bottom maps and then confirmed during exploratory ROV surveys. Sampling blocks that were 500 m wide and up to 3 km long were then created using GIS maps. Reference sites were selected based on map-based estimations of similarity in the types and amounts of rocky substrate present, proximity to one another, and depth. A stratified-random design of transects was used and the ROV recorded video while moving along a fixed transect path along the seafloor. Thus, videos collected from 500 m long transect lines were used as sampling units. Video imagery collected was analyzed to characterize substrate types present and to identify and count all demersal and epibenthic finfish and macro-invertebrates. Using a series of non-overlapping video quadrats, the distribution, relative abundance, and density of species were estimated along each transect, as described in [(Auster et al., 1997)](https://www.zotero.org/google-docs/?w2Di7g) and [(Lindholm et al., 2004)](https://www.zotero.org/google-docs/?78gaxM). All organisms were identified, enumerated, and recorded with UTC timecode and linked to ROV position and sensor files. Forward digital still photographs were used to verify species identifications where high resolution of species characteristics was required. Estimates of fish total lengths were made using paired lasers mounted on the ROV. Total length represents the distance from the tip of the snout to the most posterior part of the caudal fin without compressing the tail. Deep reef monitoring is led by the Starr lab at Moss Landing Marine Laboratories.

#### *Estimated larval settlement magnitude*

We estimated larval settlement magnitude across the MPA network as the total summed settlement of propagules (spores, eggs, larvae) entering each MPA. We used a Regional Ocean Model System (ROMS) particle tracking which simulates dispersal in three spatial dimensions (through the movement of X, Y, Z vectors) and one temporal dimension. This simulated dispersal is based on an average solution across 15 years (1999-2013) and the range spans from 100km south of CA into Baja California, Mexico, and north up through Oregon, although for this project we focused on only California’s range. Approximately 88000 “propagule” particles are released across 557 ROMS cells (365 cells in California). Larvae move hourly, but with daily averaged currents (i.e., every hour we interpolate the daily average currents from the ROMS model in space and time to find the current at each particle location, and then we move each particle with its appropriate current velocity). Landward of the 500 m depth isobath, larvae are also given a random pulse simulating tidal currents of 5 cm/s. This pulse is also given every hour in addition to the daily-averaged motion. Settlement only occurs within 10% of PLD (e.g., for PLD of 30 days: 27-33 days).

ROMS solutions were run at three “lengths of time” to simulate differing PLDs of common species’ life history: 30, 60, and 90 days. We then took an elemental average of the corresponding three PLD matrices to represent an average dispersal across common species inhabiting Rocky intertidal, Shallow Rocky Reef, 30-100m Rock, and 100-200m Rock habitats. The ROMS output can be considered a measure of connectivity among cells (locations) but should not be considered – on its own – an estimate of one cell’s contribution of propagules to other cells. This is because cells in ROMS grids are only characterized by oceanographic forcing and spatial dimensions (and vertical layers), and we simulated the release of the same number of particles from each cell. To estimate the actual settlement of a species, propagule production for donor cells and the amount of suitable habitat for receiving cells was incorporated based on the idea that propagules will scale with the amount of available habitat a species occupies in both donor and settlement locations. Specifically, across each habitat type we took the product of the donor-to-recipient cell propagule connectivity with the area of habitat in the donor cell and the area of habitat in the recipient cell. We then summed all propagule contributions coming from every donor cell (both MPAs and areas of fishing) into a recipient cell (MPAs only) to quantify the total summed settlement across all MPAs within the network.

#### *Habitat estimates*

We examined the habitat richness, habitat diversity, and total proportion of rock within MPAs using estimates of the area extent of major habitats present within the boundaries of each MPA (**Appendix S6**). Major habitats include both nearshore/offshore (0-3000 m depth) and onshore (shoreline) characteristics identified as important during the MPA planning process. Habitat richness was calculated as the number of unique habitat types within each MPA. Habitat diversity was calculated as Shannon diversity using the area of each habitat type within the MPA. Proportion rock was calculated as the total area of hard substrate (across all depths) divided by the total size of the MPA.

*Evaluating ecosystem-level performance drivers using random forests*

We used the *randomForest* package in R [(Liaw & Wiener, 2002)](https://www.zotero.org/google-docs/?w8SPdV) to fit and evaluate ecosystem-specific random forest models that predict conservation performance based on the eight evaluated MPA features. We evaluated feature importance as the mean decrease in node impurity resulting from splitting the tree on each feature and we evaluated the marginal effects of each feature on conservation performance by measuring its impact when holding the other features at their ecosystem-specific averages. Importantly, the random forest models used the most recent year of sampling for each MPA to reflect the longest duration of protection. The model was constructed using 500 trees with one third of the variables sampled at each split.

## **Appendix S2**

#### *Ecosystem-level predictors of conservation performance*

The ecosystem-specific random forest models explained a large proportion of the variation in conservation performance (log ratio effect size) across MPAs (r^2^=0.65-0.77; *SI* **Appendix S17**). However, the importance of each MPA feature varied by ecosystem. MPA age and size (area) were generally strong predictors of ecosystem-specific performance, with the surf zone and deep reef ecosystems responding particularly positively to MPA size. Local pre-implementation fisheries landings were positively correlated with conservation performance in the deep reef ecosystem, such that fish biomass responded more strongly and positively to regulatory protection. Habitat diversity and richness were also generally positively correlated with conservation performance. Interestingly, estimated settlement magnitude was important on an individual-ecosystem level, especially for the surf zone and shallow reef ecosystems, where higher settlement magnitude was correlated with greater targeted fish biomass.

###

**Appendix S4.** Observed species by ecosystem (surf zone, kelp forest, shallow reef, and deep reef) and their target status. An “X” indicates that a species was observed for a given ecosystem and empty cells indicate that a given species was not observed. Species with an “R” designation were non-resident (baitfish or pelagic species, often with high abundance but observed at low frequencies) for a given ecosystem and were removed from the analyses to avoid potential bias.

| **Scientific name** | **Target status** | **Surf zone** | **Kelp forest** | **Shallow reef** | **Deep reef** |
| --- | --- | --- | --- | --- | --- |
| *Agonidae spp* | Non-targeted |  |  |  | X |
| *Alopias vulpinus* | Targeted |  | R | R |  |
| *Ammodytes hexapterus* | Targeted |  |  |  | X |
| *Amphistichus argenteus* | Targeted | X | X |  | X |
| *Amphistichus koelzi* | Targeted | X |  |  |  |
| *Amphistichus rhodoterus* | Targeted | X |  |  |  |
| *Anarrhichthys ocellatus* | Non-targeted |  | X | X | X |
| *Anisotremus davidsonii* | Targeted |  | X | X |  |
| *Anoplagonus inermis* | Non-targeted |  | X |  |  |
| *Anoplopoma fimbria* | Targeted |  |  |  | X |
| *Apodichthys flavidus* | Non-targeted |  | X |  |  |
| *Apogon guadalupensis* | Non-targeted |  | X |  |  |
| *Apristurus brunneus* | Non-targeted |  |  |  | R |
| *Artedius harringtoni* | Non-targeted |  |  | X |  |
| *Atherinidae spp* | Targeted | X |  |  |  |
| *Atherinops affinis* | Targeted | X |  | X |  |
| *Atherinopsidae spp* | Non-targeted |  | X | X |  |
| *Atherinopsis californiensis* | Targeted |  |  | X |  |
| *Atractoscion nobilis* | Targeted | X | X | X |  |
| *Aulorhynchus flavidus* | Non-targeted | X | X | R | X |
| *Balistes polylepis* | Non-targeted |  | X |  |  |
| *Bathymasteridae spp* | Non-targeted |  | X |  |  |
| *Beringraja binoculata* | Targeted |  | X |  | X |
| *Beringraja rhina* | Targeted |  |  |  | X |
| *Beringraja stellulata* | Targeted |  | X | X | X |
| *Bothidae spp* | Targeted |  | X |  |  |
| *Brachygenys californiensis* | Targeted |  | X | X |  |
| *Brachyistius frenatus* | Non-targeted | X | X |  | X |
| *Caliraja spp* | Targeted |  |  |  | X |
| *Carcharhinus obscurus* | Targeted |  | R |  |  |
| *Caulolatilus princeps* | Targeted |  | X | X | X |
| *Cebidichthys violaceus* | Targeted |  | X |  |  |
| *Cephaloscyllium ventriosum* | Non-targeted |  | X |  | X |
| *Cheilotrema saturnum* | Targeted | X | X |  |  |
| *Chromis punctipinnis* | Non-targeted |  | X | X | X |
| *Citharichthys sordidus* | Targeted |  | X | X | X |
| *Citharichthys spp* | Targeted |  | X | X |  |
| *Citharichthys stigmaeus* | Targeted | X | X | X |  |
| *Citharichthys xanthostigma* | Targeted |  |  | X |  |
| *Clinidae spp* | Non-targeted | X |  |  |  |
| *Clupea pallasii* | Targeted | X |  |  |  |
| *Cottidae spp* | Non-targeted |  |  |  | X |
| *Cryptacanthodes giganteus* | Non-targeted |  | X |  |  |
| *Cymatogaster aggregata* | Targeted | X | X |  | X |
| *Decapterus macarellus* | Targeted |  | R |  |  |
| *Embiotoca jacksoni* | Targeted | X | X |  | X |
| *Embiotoca lateralis* | Targeted | X | X | X | X |
| *Embiotocidae spp* | Targeted |  | X |  | X |
| *Engraulis mordax* | Targeted | X | R | R |  |
| *Enophrys bison* | Non-targeted |  | X | X | X |
| *Enophrys taurina* | Non-targeted |  |  | X |  |
| *Entosphenus tridentatus* | Non-targeted |  |  |  | X |
| *Eopsetta jordani* | Targeted |  |  | X | X |
| *Eptatretus stoutii* | Targeted |  |  |  | X |
| *Ernogrammus walkeri* | Non-targeted |  | X |  |  |
| *Fundulus parvipinnis* | Non-targeted | X |  |  |  |
| *Gadidae spp* | Non-targeted |  |  |  | X |
| *Galeorhinus galeus* | Targeted |  | R | R |  |
| *Genyonemus lineatus* | Targeted | X |  | X |  |
| *Gibbonsia metzi* | Non-targeted | X |  |  | X |
| *Gibbonsia montereyensis* | Non-targeted | X |  |  |  |
| *Gibbonsia spp* | Non-targeted | X |  |  |  |
| *Girella nigricans* | Targeted | X | X | X | X |
| *Glyptocephalus zachirus* | Targeted |  |  |  | X |
| *Gobiesox maeandricus* | Non-targeted |  | X |  |  |
| *Gobiidae spp* | Non-targeted |  |  |  | X |
| *Gymnothorax mordax* | Non-targeted |  | X |  | X |
| *Halichoeres semicinctus* | Non-targeted |  | X | X | X |
| *Hemilepidotus hemilepidotus* | Non-targeted |  | X |  |  |
| *Hemilepidotus spinosus* | Non-targeted |  | X |  |  |
| *Heterodontus francisci* | Non-targeted |  | X |  | X |
| *Heterostichus rostratus* | Non-targeted | X | X |  |  |
| *Hexagrammidae spp* | Targeted |  |  |  | X |
| *Hexagrammos decagrammus* | Targeted |  | X | X | X |
| *Hexagrammos lagocephalus* | Targeted |  | X | X | X |
| *Hexagrammos spp* | Targeted |  | X |  |  |
| *Hexanchus griseus* | Targeted |  | R |  | R |
| *Hippoglossina stomata* | Targeted |  |  | X |  |
| *Hippoglossus stenolepis* | Targeted |  |  | X | X |
| *Hydrolagus colliei* | Non-targeted |  |  |  | X |
| *Hyperprosopon anale* | Targeted | X | X |  |  |
| *Hyperprosopon argenteum* | Targeted | X | X |  |  |
| *Hyperprosopon ellipticum* | Targeted | X | X |  |  |
| *Hypomesus pretiosus* | Targeted | X |  |  |  |
| *Hypsurus caryi* | Targeted | X | X |  | X |
| *Hypsypops rubicundus* | Non-targeted |  | X | X | X |
| *Kyphosus azureus* | Targeted | X | X |  |  |
| *Leiocottus hirundo* | Non-targeted |  | X |  |  |
| *Lepidopsetta bilineata* | Targeted |  |  | X | X |
| *Leptocottus armatus* | Non-targeted | X | X |  | X |
| *Lethops connectens* | Non-targeted |  | X |  |  |
| *Lycodes pacificus* | Targeted |  |  |  | X |
| *Lyopsetta exilis* | Targeted |  |  |  | X |
| *Lythrypnus dalli* | Non-targeted |  |  |  | X |
| *Macrouridae spp* | Non-targeted |  |  |  | X |
| *Medialuna californiensis* | Targeted | X | X | X | X |
| *Menticirrhus undulatus* | Targeted | X |  |  |  |
| *Merluccius productus* | Targeted |  |  |  | X |
| *Micrometrus aurora* | Non-targeted | X |  |  |  |
| *Micrometrus minimus* | Non-targeted | X | X |  |  |
| *Microstomus pacificus* | Targeted |  |  |  | X |
| *Mola mola* | Non-targeted |  | R |  | R |
| *Morone saxatilis* | Targeted | X |  |  |  |
| *Mugil cephalus* | Targeted | X |  |  |  |
| *Myliobatis californica* | Targeted | X | X | X | X |
| *Neoclinus blanchardi* | Non-targeted |  | X | X |  |
| *Notorynchus cepedianus* | Targeted |  | R |  |  |
| *Oncorhynchus tshawytscha* | Targeted |  |  | R |  |
| *Ophiodon elongatus* | Targeted |  | X | X | X |
| *Osmeridae spp* | Targeted |  |  |  | X |
| *Oxyjulis californica* | Non-targeted |  | X | X | X |
| *Oxylebius pictus* | Non-targeted |  | X | X | X |
| *Paralabrax clathratus* | Targeted | X | X | X | X |
| *Paralabrax maculatofasciatus* | Targeted |  | X |  |  |
| *Paralabrax nebulifer* | Targeted |  | X | X |  |
| *Paralichthyidae spp* | Targeted | X |  |  | X |
| *Paralichthys californicus* | Targeted | X | X | X | X |
| *Parophrys vetulus* | Targeted |  |  |  | X |
| *Pegusa lascaris* | Targeted |  |  |  | X |
| *Phanerodon atripes* | Non-targeted |  | X | X | X |
| *Phanerodon furcatus* | Targeted | X | X |  | X |
| *Phanerodon vacca* | Targeted | X | X | X | X |
| *Pholidae spp* | Non-targeted | X | X |  |  |
| *Platichthys stellatus* | Targeted | X |  |  | X |
| *Platyrhinoidis triseriata* | Non-targeted | X | X |  |  |
| *Pleuronectidae spp* | Targeted |  | X |  | X |
| *Pleuronichthys coenosus* | Non-targeted |  | X |  |  |
| *Pleuronichthys ritteri* | Non-targeted |  |  |  | X |
| *Porichthys notatus* | Non-targeted |  | X |  | X |
| *Prionace glauca* | Targeted |  | R |  |  |
| *Prognathodes falcifer* | Non-targeted |  | X |  |  |
| *Psettichthys melanostictus* | Targeted | X |  | X |  |
| *Pseudobatos productus* | Targeted | X | X |  |  |
| *Rajidae spp* | Targeted |  |  |  | X |
| *Rathbunella alleni* | Non-targeted |  | X |  |  |
| *Rathbunella hypoplecta* | Non-targeted |  | X |  | X |
| *Rhacochilus toxotes* | Targeted |  | X | X | X |
| *Rhamphocottus richardsonii* | Non-targeted |  | X |  |  |
| *Rhinogobiops nicholsii* | Non-targeted |  | X |  | X |
| *Roncador stearnsii* | Targeted | X |  |  |  |
| *Ronquilus jordani* | Non-targeted |  | X |  |  |
| *Sarda chiliensis* | Targeted |  | R | X |  |
| *Sardinops sagax* | Targeted | X | R | R |  |
| *Sciaenidae spp* | Targeted |  |  |  | X |
| *Scomber japonicus* | Targeted |  | R | X |  |
| *Scombridae spp* | Targeted |  |  | X |  |
| *Scorpaena guttata* | Targeted |  | X | X | X |
| *Scorpaenichthys marmoratus* | Targeted | X | X | X | X |
| *Scorpaenidae spp* | Targeted |  |  |  | X |
| *Scorpaenodes xyris* | Non-targeted |  | X |  |  |
| *Sebastes atrovirens* | Targeted |  | X | X | X |
| *Sebastes auriculatus* | Targeted |  | X | X | X |
| *Sebastes aurora* | Targeted |  |  |  | X |
| *Sebastes babcocki* | Non-targeted |  |  |  | X |
| *Sebastes borealis* | Targeted |  |  |  | X |
| *Sebastes carnatus* | Targeted |  | X | X | X |
| *Sebastes caurinus* | Targeted |  | X | X | X |
| *Sebastes chlorostictus* | Targeted |  |  |  | X |
| *Sebastes chrysomelas* | Targeted |  | X | X | X |
| *Sebastes constellatus* | Targeted |  |  | X | X |
| *Sebastes crameri* | Targeted |  |  |  | X |
| *Sebastes dallii* | Targeted |  | X | X | X |
| *Sebastes diaconus* | Targeted |  |  | X |  |
| *Sebastes diploproa* | Targeted |  | X |  | X |
| *Sebastes elongatus* | Targeted |  |  |  | X |
| *Sebastes ensifer* | Targeted |  |  |  | X |
| *Sebastes entomelas* | Targeted |  | X | X | X |
| *Sebastes flavidus* | Targeted |  |  | X | X |
| *Sebastes goodei* | Targeted |  |  |  | X |
| *Sebastes helvomaculatus* | Non-targeted |  |  |  | X |
| *Sebastes hopkinsi* | Targeted |  | X | X | X |
| *Sebastes jordani* | Targeted |  |  |  | X |
| *Sebastes lentiginosus* | Targeted |  |  | X | X |
| *Sebastes levis* | Targeted |  |  |  | X |
| *Sebastes maliger* | Targeted |  | X | X | X |
| *Sebastes melanops* | Targeted |  | X | X | X |
| *Sebastes melanostomus* | Targeted |  |  |  | X |
| *Sebastes miniatus* | Targeted |  | X | X | X |
| *Sebastes mystinus* | Targeted |  | X | X | X |
| *Sebastes nebulosus* | Targeted |  | X | X | X |
| *Sebastes nigrocinctus* | Targeted |  |  | X | X |
| *Sebastes ovalis* | Targeted |  |  |  | X |
| *Sebastes paucispinis* | Targeted | X | X | X | X |
| *Sebastes pinniger* | Targeted |  | X | X | X |
| *Sebastes rastrelliger* | Targeted | X | X | X |  |
| *Sebastes rosaceus* | Targeted |  | X | X | X |
| *Sebastes rosenblatti* | Targeted |  |  |  | X |
| *Sebastes ruberrimus* | Targeted |  |  | X | X |
| *Sebastes rubrivinctus* | Targeted |  | X |  | X |
| *Sebastes rufus* | Targeted |  |  |  | X |
| *Sebastes saxicola* | Targeted |  | X |  | X |
| *Sebastes semicinctus* | Targeted |  | X |  | X |
| *Sebastes serranoides* | Targeted |  |  | X | X |
| *Sebastes serriceps* | Targeted |  | X | X | X |
| *Sebastes simulator* | Targeted |  |  |  | X |
| *Sebastes spp* | Targeted | X | X | X | X |
| *Sebastes umbrosus* | Targeted |  | X | X | X |
| *Sebastes wilsoni* | Non-targeted |  |  |  | X |
| *Sebastidae spp* | Targeted |  |  |  | X |
| *Sebastolobus alascanus* | Targeted |  |  |  | X |
| *Semicossyphus pulcher* | Targeted |  | X | X | X |
| *Seriola lalandi* | Targeted |  | X | X | X |
| *Seriphus politus* | Targeted | X |  |  |  |
| *Sphyraena argentea* | Targeted |  | X | X |  |
| *Squalus acanthias* | Targeted |  | X | X |  |
| *Squatina californica* | Targeted |  | X |  | X |
| *Stellerina xyosterna* | Non-targeted | X |  |  |  |
| *Stereolepis gigas* | Non-targeted |  | X | X | X |
| *Stichaeidae spp* | Non-targeted |  | X |  |  |
| *Syngnathus californiensis* | Non-targeted | X |  |  |  |
| *Syngnathus leptorhynchus* | Non-targeted | X |  |  |  |
| *Syngnathus spp* | Non-targeted | X | X |  |  |
| *Synodus lucioceps* | Non-targeted |  | X | X | X |
| *Tetronarce californica* | Non-targeted |  | X |  | X |
| *Thaleichthys pacificus* | Targeted |  | R |  |  |
| *Trachurus symmetricus* | Targeted |  | X | X |  |
| *Triakis semifasciata* | Targeted | X | X |  |  |
| *Ulvicola sanctaerosae* | Non-targeted |  | X |  |  |
| *Umbrina roncador* | Targeted | X |  | X |  |
| *Urobatis halleri* | Targeted | X | X |  |  |
| *Xystreurys liolepis* | Targeted |  |  | X |  |
| *Zalembius rosaceus* | Non-targeted |  | X |  | X |
| *Zaniolepis frenata* | Non-targeted |  |  |  | X |
| *Zaniolepis latipinnis* | Non-targeted |  |  |  | X |
| *Zaniolepis spp* | Non-targeted |  |  |  | X |
| *Zapteryx exasperata* | Targeted |  | X |  |  |
| *Zoarcidae spp* | Non-targeted | X |  |  | X |

**Appendix S5.** MPA features used to predict conservation performance. California Department of Fish and Wildlife (CDFW); Regional Ocean Modeling System (ROMS); Environmental Sensitivity Index (ESI).

| **Category** | **Feature** | **Source** | **Details** |
| --- | --- | --- | --- |
| MPA feature | MPA age (yr) | CDFW MPA GIS file |  |
| MPA feature | MPA size (km^2^) | CDFW MPA GIS file |  |
| MPA feature | Regulatory status | CDFW MPA GIS file |  |
| Habitat | Settlement magnitude |  | Simulated using ROMS (see supplementary *Methods*) |
| Habitat | Habitat richness | CDFW bottom substrate and ESI shoreline | Number of unique habitat types |
| Habitat | Habitat diversity | CDFW bottom substrate and ESI shoreline | Shannon diversity using area of each habitat type |
| Habitat | Proportion of rock bottom | CDFW bottom substrate | Total hard substrate divided by MPA size |
| Human | Pre-MPA fishing pressure | CDFW fish tickets | Calculated at the block-level using 10 years of landings data preceding the oldest MPA in the block. |
| Biological | Species traits | Shallow reef (CCFRP), kelp forest, surf zone, deep reef, FishBase | Each ecosystem monitoring program conducted a literature review of species traits. We created a standardized table using these traits, and then identified missing traits from FishBase. |

**Appendix S6.** Habitat types and data sources used to estimate habitat diversity, habitat richness, and proportion of rock within each MPA.

| **Habitat Type** | **Data Information** |
| --- | --- |
| Hard substrate (0-30m) | High resolution (2m to 10m) multibeam mapping, mostly from the [California Seafloor Mapping Project](https://csumb.edu/undersea/seafloor-maps/). Area totals calculated from a vector file. Depth information from the high resolution bathymetry data where available. Small mapping gaps filled in through interpolation and added to the total. |
| Hard substrate (30-100m) |  |
| Hard substrate (100-200m) |  |
| Hard substrate (200-3000m) |  |
| Soft substrate (0-30m) |  |
| Soft substrate (30-100m) |  |
| Soft substrate (100-200m) |  |
| Soft substrate (200-3000m) |  |
| Kelp canopy (0-30m) | Data from CDFW kelp overflights (14 years; '89, '99, '02-'06, '08-'10, '13-'16), composite of all available data for maximum canopy extent.(Saarman 2020, unpublished). Captures both giant and bull kelp and covers the whole coast of California. |
| Coastal marsh | Data from [NOAA ESI shoreline](https://response.restoration.noaa.gov/resources/environmental-sensitivity-index-esi-maps) data, using the 2010 update for southern California. Source data has up to 3 classifications for each coastal segment (landward, seaward1, seaward2), length totals reflect all of these classifications, but do not double-count (for example landward is gravel beach, seaward1 is fine-grained beach, this segment counted just once as beach). Linear estimates were converted to area using median beach widths. |
| Tidal flats |  |
| Hardened/armored shoreline |  |
| Sandy beach |  |
| Rocky intertidal |  |

**Appendix S7.** List of *de facto* MPAs for each ecosystem. MPA type is the state-designated status of the MPA, and the *de facto* status is indicated for each ecosystem. An MPA was designated as a *de facto* no-take MPA for a particular ecosystem if any allowed partial-take was unlikely to affect the species that reside in that particular ecosystem (e.g., take of salmon in an MPA is unlikely to affect any of our four focal ecosystems; Smith et al. 2023). Empty cells indicate that particular MPA was not sampled within a given ecosystem.

| **MPA name** | **MPA type** | **Surf zone** | **Kelp forest** | **Shallow reef** | **Deep reef** |
| --- | --- | --- | --- | --- | --- |
| Campus Point SMCA | SMCA | SMR | SMR |  | SMR |
| White Rock SMCA | SMCA |  | SMR |  |  |
| Point Vicente SMCA | SMCA |  | SMR |  |  |
| Blue Cavern Onshore SMCA | SMCA |  | SMR |  |  |
| Abalone Cove SMCA | SMCA |  | SMR |  |  |
| Farnsworth Onshore SMCA | SMCA |  | SMR |  |  |
| Point Dume SMCA | SMCA |  | SMR |  |  |
| Swami's SMCA | SMCA |  | SMR | SMR |  |
| Piedras Blancas SMCA | SMCA |  |  |  | SMR |
| Point Sur SMCA | SMCA |  |  |  | SMR |
| Southeast Farallon Island SMCA | SMCA |  |  |  | SMR |
| Point Arena SMCA | SMCA |  |  |  | SMR |
| Portuguese Ledge SMCA | SMCA |  |  |  | SMR |
| Big Creek SMCA | SMCA |  |  |  | SMR |

**Appendix S8.** Sampling years by ecosystem for MPAs with paired reference sites. Empty cells indicate that a particular MPA was not sampled within a given ecosystem.

| **MPA name** | **Surf zone** | **Kelp forest** | **Shallow reef** | **Deep reef** |
| --- | --- | --- | --- | --- |
| Abalone Cove SMCA |  | 2011, 2013, 2015, 2016, 2017, 2018, 2019, 2020 |  |  |
| Anacapa Island SMCA |  | 2004, 2005, 2006, 2007, 2008, 2009 |  |  |
| Anacapa Island SMR |  | 2004, 2005, 2006, 2007, 2008, 2009, 2010, 2011, 2012, 2014, 2015, 2016, 2017, 2018, 2019, 2020 | 2017, 2018, 2019, 2020 |  |
| Asilomar SMR | 2019, 2020 | 2007, 2008, 2011 |  | 2007, 2008 |
| Año Nuevo SMR | 2019, 2020 |  | 2007, 2008, 2009, 2010, 2011, 2012, 2013, 2014, 2015, 2016, 2017, 2018, 2019, 2020 | 2015, 2019 |
| Begg Rock SMR |  | 2009, 2013 |  |  |
| Big Creek SMR |  | 2001, 2002, 2003, 2004, 2005, 2006, 2007, 2008, 2009, 2010, 2011, 2015 |  | 2016 |
| Blue Cavern Onshore SMCA |  | 2004, 2011, 2012, 2019, 2020 |  |  |
| Bodega Head SMR |  |  | 2017, 2018, 2019, 2020 | 2015, 2019 |
| Cambria SMCA |  | 2003, 2004, 2005, 2007, 2008 |  |  |
| Campus Point SMCA | 2019, 2020 | 2009, 2010, 2011, 2012, 2013, 2014, 2015, 2016, 2017, 2018, 2019, 2020 |  | 2014, 2019 |
| Carmel Bay SMCA |  | 2001, 2002, 2003, 2004, 2005, 2006, 2007, 2008, 2009, 2010, 2011, 2013, 2014, 2015, 2016, 2017, 2018, 2019, 2020 |  |  |
| Carrington Point SMR |  | 2003, 2004, 2005, 2007, 2008 | 2017, 2018, 2019, 2020 | 2005, 2006, 2007, 2009, 2014, 2019 |
| Cat Harbor SMCA |  | 2004, 2005, 2011, 2012, 2019, 2020 |  |  |
| Crystal Cove SMCA |  | 2011, 2012, 2019, 2020 |  |  |
| Del Mar Landing SMR |  | 2010, 2011 |  |  |
| Farnsworth Onshore SMCA |  | 2011, 2012, 2019 |  |  |
| Gull Island SMR |  | 2003, 2004, 2005, 2006, 2007, 2008, 2009, 2010, 2011, 2012, 2014, 2015, 2016, 2017, 2018, 2019, 2020 |  | 2014, 2019 |
| Harris Point SMR |  | 2003, 2004, 2005, 2006, 2007, 2008, 2009, 2010, 2011, 2012, 2014, 2016, 2018, 2019, 2020 |  | 2005, 2006, 2007, 2009, 2014, 2019 |
| Laguna Beach SMR | 2019, 2020 |  | 2017 |  |
| Long Point SMR |  | 2011, 2012, 2019, 2020 |  |  |
| Lovers Point - Julia Platt SMR |  | 2000, 2001, 2002, 2003, 2004, 2005, 2006, 2007, 2008, 2009, 2011, 2012, 2013, 2014, 2015, 2016, 2017, 2018, 2019, 2020 |  |  |
| Matlahuayl SMR | 2019, 2020 | 2011, 2012, 2019, 2020 |  |  |
| Naples SMCA |  | 2009, 2010, 2011, 2012, 2013, 2014, 2015, 2016, 2017, 2018, 2019, 2020 |  |  |
| Natural Bridges SMR | 2019, 2020 | 2000, 2001, 2002, 2003, 2004, 2005, 2006, 2007, 2008, 2009, 2010, 2011 |  |  |
| Painted Cave SMCA |  | 2003, 2004, 2005, 2006, 2007, 2008, 2009, 2010, 2011, 2012, 2014, 2015, 2016, 2017, 2018, 2019, 2020 |  |  |
| Piedras Blancas SMR |  | 2003, 2004, 2005, 2006, 2007, 2008 | 2008, 2009, 2010, 2011, 2012, 2013, 2014, 2016, 2017, 2018, 2019, 2020 |  |
| Pillar Point SMCA |  |  |  | 2015 |
| Point Arena SMR |  | 2011 |  |  |
| Point Buchon SMR |  | 2007, 2008, 2009, 2010, 2011, 2016, 2017, 2018, 2019, 2020 | 2007, 2008, 2009, 2010, 2011, 2012, 2013, 2014, 2015, 2016, 2017, 2018, 2019, 2020 | 2008, 2009, 2016, 2019 |
| Point Cabrillo SMR |  | 2014, 2015, 2017, 2018, 2019 |  |  |
| Point Conception SMR | 2019, 2020 | 2000, 2001, 2002, 2003, 2004, 2005, 2006, 2007, 2009, 2010, 2011, 2012 | 2018 | 2014, 2019 |
| Point Dume SMCA |  | 2010, 2011, 2012, 2013, 2019, 2020 |  |  |
| Point Dume SMR | 2019, 2020 | 2008, 2019, 2020 |  |  |
| Point Lobos SMR | 2019, 2020 | 2006, 2007, 2008, 2009, 2010, 2011, 2012, 2013, 2014, 2015, 2016, 2017, 2018, 2019, 2020 | 2007, 2008, 2009, 2010, 2011, 2012, 2013, 2014, 2015, 2016, 2017, 2018, 2019, 2020 | 2008, 2009, 2016, 2019 |
| Point Reyes SMR | 2019, 2020 |  |  |  |
| Point St. George Reef Offshore SMCA |  |  |  | 2014 |
| Point Sur SMCA |  |  |  | 2008 |
| Point Sur SMR |  | 2005, 2006, 2007, 2008, 2010, 2011, 2017, 2019, 2020 |  |  |
| Point Vicente SMCA |  | 2004, 2007, 2008, 2009, 2010, 2011, 2012, 2013, 2014, 2015, 2016, 2017, 2018, 2019, 2020 |  |  |
| Portuguese Ledge SMCA |  |  |  | 2016, 2019 |
| Pyramid Point SMCA |  | 2018 |  |  |
| Reading Rock SMCA | 2019, 2020 |  |  |  |
| Salt Point SMCA |  | 2010, 2011 |  |  |
| Samoa SMCA | 2019, 2020 |  |  |  |
| Santa Barbara Island SMR |  | 2004, 2005, 2006, 2007, 2008, 2011, 2013, 2019 |  |  |
| Saunders Reef SMCA |  | 2010, 2011, 2016, 2017, 2018, 2019, 2020 |  |  |
| Scorpion SMR |  | 2004, 2005, 2006, 2007, 2008, 2009, 2010, 2011, 2012, 2013, 2014, 2015, 2016, 2017, 2018, 2019, 2020 |  |  |
| Sea Lion Cove SMCA |  | 2010, 2011 |  |  |
| Sea Lion Gulch SMR |  |  |  | 2014 |
| South Cape Mendocino SMR |  |  | 2017, 2018, 2019, 2020 |  |
| South La Jolla SMR |  | 2011, 2012, 2019, 2020 | 2017, 2018, 2019, 2020 |  |
| South Point SMR |  | 2004, 2005, 2006, 2007, 2008, 2009, 2010, 2011, 2012, 2013, 2014, 2015, 2016, 2017, 2018, 2019, 2020 |  | 2005, 2006, 2007, 2009, 2014, 2019 |
| Southeast Farallon Island SMR |  |  | 2017, 2018 | 2011 |
| Stewarts Point SMR |  | 2010, 2011, 2017, 2018, 2019 | 2017, 2018, 2019, 2020 |  |
| Swami's SMCA |  | 2011, 2012, 2019 | 2017, 2018, 2019, 2020 |  |
| Ten Mile SMR | 2019, 2020 | 2014, 2015, 2018, 2019, 2020 | 2017, 2018, 2019, 2020 | 2014 |
| Vandenberg SMR |  | 2000, 2001, 2002, 2003, 2004, 2005, 2006, 2008 |  |  |
| White Rock SMCA |  | 2003, 2004, 2005, 2006, 2007, 2008, 2009, 2011 |  |  |

**Appendix S9.** Network-level meta-analysis results pooled across the entire network of MPAs, ecosystems, and regions. The p-values (p) in red text indicates statistically significant relationships.

| **MPA type** | **Allowed take** | **Target status** | **Effect size** | **Standard error** | **P-value** | **95% lower** | **95% upper** | ***n* MPA-Ecosystem pairs** | **Tau-2** | **Q** |
| --- | --- | --- | --- | --- | --- | --- | --- | --- | --- | --- |
| SMCA | Partial-take | Targeted | 0.593 | 0.315 | 0.059 | -0.024 | 1.21 | 15 | 1.241 | 120.23 |
| SMR | No-take | Targeted | 0.497 | 0.096 | *<0.001* | 0.31 | 0.685 | 78 | 0.569 | 1000.213 |
| SMCA | Partial-take | Non-targeted | 0.225 | 0.319 | 0.48 | -0.4 | 0.85 | 14 | 1.307 | 5745.747 |
| SMR | No-take | Non-targeted | 0.167 | 0.085 | *0.049* | 0.001 | 0.333 | 62 | 0.365 | 1774.773 |

**Appendix S10.** Regional-level meta-analysis results from meta-analyses pooled across MPAs and ecosystems within a region. The p-values (p) in red text indicates statistically significant relationships.

| **Region** | **MPA type** | **Allowed take** | **Target status** | **Effect size** | **Standard error** | **P-value** | **95% lower** | **95% upper** | ***n* MPA-Ecosystem pairs** | **Tau-2** | **Q** |
| --- | --- | --- | --- | --- | --- | --- | --- | --- | --- | --- | --- |
| North Coast | SMCA | Partial-take | Targeted | 1.901 | 0.912 | *0.037* | 0.115 | 3.688 | 4 | 2.967 | 17.48 |
| North Coast | SMR | No-take | Targeted | 0.213 | 0.193 | 0.27 | -0.165 | 0.592 | 7 | 0.162 | 17.592 |
| North Coast | SMCA | Partial-take | Non-targeted | 1.22 | 0.721 | 0.09 | -0.192 | 2.633 | 4 | 1.849 | 50.963 |
| North Coast | SMR | No-take | Non-targeted | 0.112 | 0.245 | 0.647 | -0.368 | 0.593 | 5 | 0.276 | 621.643 |
| North Central Coast | SMCA | Partial-take | Targeted | 0.114 | 0.349 | 0.743 | -0.57 | 0.798 | 4 | 0.222 | 6.077 |
| North Central Coast | SMR | No-take | Targeted | 0.582 | 0.244 | *0.017* | 0.104 | 1.061 | 9 | 0.428 | 86.178 |
| North Central Coast | SMCA | Partial-take | Non-targeted | 0.022 | 0.38 | 0.953 | -0.722 | 0.767 | 3 | 0.432 | 560.025 |
| North Central Coast | SMR | No-take | Non-targeted | 0.252 | 0.145 | 0.082 | -0.032 | 0.536 | 5 | 0.098 | 37.954 |
| Central Coast | SMCA | Partial-take | Targeted | -0.271 | 0.331 | 0.414 | -0.92 | 0.379 | 2 | 0.158 | 3.432 |
| Central Coast | SMR | No-take | Targeted | 0.356 | 0.154 | *0.021* | 0.054 | 0.659 | 25 | 0.451 | 168.506 |
| Central Coast | SMCA | Partial-take | Non-targeted | -0.087 | 0.447 | 0.845 | -0.964 | 0.79 | 2 | 0.352 | 8.177 |
| Central Coast | SMR | No-take | Non-targeted | 0.224 | 0.124 | 0.07 | -0.019 | 0.466 | 21 | 0.257 | 228.277 |
| South Coast | SMCA | Partial-take | Targeted | 0.376 | 0.511 | 0.462 | -0.625 | 1.378 | 5 | 1.149 | 41.689 |
| South Coast | SMR | No-take | Targeted | 0.641 | 0.156 | *<0.001* | 0.335 | 0.946 | 37 | 0.735 | 620.309 |
| South Coast | SMCA | Partial-take | Non-targeted | -0.291 | 0.534 | 0.586 | -1.338 | 0.757 | 5 | 1.289 | 28.022 |
| South Coast | SMR | No-take | Non-targeted | 0.139 | 0.149 | 0.352 | -0.154 | 0.431 | 31 | 0.565 | 313.5 |

**Appendix S11.** Ecosystem-level meta-analysis results from meta-analyses pooled across MPAs within an ecosystem and target status. The p-values (p) in red text indicates statistically significant relationships.

| **Ecosystem** | **MPA type** | **Allowed take** | **Target status** | **Effect size** | **Standard error** | **P-value** | **95% lower** | **95% upper** | ***n* MPAs** | **Tau-2** | **Q** |
| --- | --- | --- | --- | --- | --- | --- | --- | --- | --- | --- | --- |
| Surf zone | SMCA | Partial-take | Targeted | 2.709 | 2.238 | 0.226 | -1.677 | 7.095 | 2 | 9.352 | 14.995 |
| Surf zone | SMR | No-take | Targeted | 0.486 | 0.282 | 0.085 | -0.066 | 1.038 | 11 | 0.653 | 61.019 |
| Surf zone | SMCA | Partial-take | Non-targeted | 1.951 | 1.022 | 0.056 | -0.052 | 3.954 | 2 | 1.896 | 10.332 |
| Surf zone | SMR | No-take | Non-targeted | 0.104 | 0.251 | 0.678 | -0.388 | 0.596 | 11 | 0.628 | 88.295 |
| Kelp forest | SMCA | Partial-take | Targeted | 0.259 | 0.282 | 0.357 | -0.293 | 0.811 | 11 | 0.681 | 86.582 |
| Kelp forest | SMR | No-take | Targeted | 0.479 | 0.164 | *0.004* | 0.157 | 0.801 | 35 | 0.761 | 663.656 |
| Kelp forest | SMCA | Partial-take | Non-targeted | -0.221 | 0.233 | 0.344 | -0.678 | 0.237 | 10 | 0.444 | 498.352 |
| Kelp forest | SMR | No-take | Non-targeted | 0.139 | 0.122 | 0.255 | -0.1 | 0.377 | 35 | 0.424 | 405.785 |
| Shallow reef | SMR | No-take | Targeted | 0.833 | 0.143 | *<0.001* | 0.553 | 1.114 | 15 | 0.272 | 137.609 |
| Deep reef | SMCA | Partial-take | Targeted | 0.898 | 0.415 | *0.031* | 0.084 | 1.712 | 2 | 0.232 | 2.985 |
| Deep reef | SMR | No-take | Targeted | 0.185 | 0.163 | 0.254 | -0.133 | 0.504 | 17 | 0.296 | 54.658 |
| Deep reef | SMCA | Partial-take | Non-targeted | 0.948 | 0.409 | *0.021* | 0.146 | 1.749 | 2 | 0.333 | 282.304 |
| Deep reef | SMR | No-take | Non-targeted | 0.276 | 0.117 | *0.018* | 0.048 | 0.505 | 16 | 0.162 | 796.162 |

**Appendix S12.** Ecosystem-level meta-analysis results by region. The p-values (p) in red text indicates statistically significant relationships.

| **Ecosystem** | **Region** | **MPA type** | **Allowed take** | **Target status** | **Effect size** | **Standard error** | **P-value** | **95% lower** | **95% upper** | ***n* MPAs** | **Tau-2** | **Q** |
| --- | --- | --- | --- | --- | --- | --- | --- | --- | --- | --- | --- | --- |
| Surf zone | North | SMCA | Partial-take | Targeted | 2.709 | 2.238 | 0.226 | -1.677 | 7.095 | 2 | 9.352 | 14.995 |
| Surf zone | North | SMR | No-take | Targeted | 0.613 | 0.087 | *<0.001* | 0.443 | 0.782 | 1 | 0 | 0 |
| Surf zone | North | SMCA | Partial-take | Non-targeted | 1.951 | 1.022 | 0.056 | -0.052 | 3.954 | 2 | 1.896 | 10.332 |
| Surf zone | North | SMR | No-take | Non-targeted | 0.785 | 0.171 | *<0.001* | 0.449 | 1.121 | 1 | 0 | 0 |
| Surf zone | North Central | SMR | No-take | Targeted | 1.053 | 0.454 | *0.02* | 0.162 | 1.944 | 1 | 0 | 0 |
| Surf zone | North Central | SMR | No-take | Non-targeted | -0.525 | 0.216 | *0.015* | -0.949 | -0.101 | 1 | 0 | 0 |
| Surf zone | Central | SMR | No-take | Targeted | -0.143 | 0.377 | 0.704 | -0.883 | 0.596 | 4 | 0.295 | 6.943 |
| Surf zone | Central | SMR | No-take | Non-targeted | -0.057 | 0.703 | 0.935 | -1.435 | 1.32 | 4 | 1.897 | 52.276 |
| Surf zone | South | SMR | No-take | Targeted | 0.789 | 0.486 | 0.104 | -0.163 | 1.742 | 5 | 0.958 | 36.967 |
| Surf zone | South | SMR | No-take | Non-targeted | 0.173 | 0.167 | 0.3 | -0.155 | 0.502 | 5 | 0.082 | 10.228 |
| Kelp forest | North | SMCA | Partial-take | Targeted | 1.199 | 0.22 | *<0.001* | 0.768 | 1.631 | 1 | 0 | 0 |
| Kelp forest | North | SMR | No-take | Targeted | -0.429 | 0.276 | 0.12 | -0.969 | 0.112 | 2 | 0 | 0.064 |
| Kelp forest | North | SMCA | Partial-take | Non-targeted | -0.731 | 0.788 | 0.354 | -2.275 | 0.813 | 1 | 0 | 0 |
| Kelp forest | North | SMR | No-take | Non-targeted | 0.322 | 0.018 | *<0.001* | 0.287 | 0.357 | 2 | 0 | 0.003 |
| Kelp forest | North Central | SMCA | Partial-take | Targeted | -0.32 | 0.286 | 0.263 | -0.881 | 0.241 | 3 | 0 | 1.681 |
| Kelp forest | North Central | SMR | No-take | Targeted | 0.687 | 0.716 | 0.338 | -0.718 | 2.091 | 3 | 1.359 | 15.067 |
| Kelp forest | North Central | SMCA | Partial-take | Non-targeted | -0.234 | 0.485 | 0.629 | -1.184 | 0.716 | 2 | 0.469 | 460.733 |
| Kelp forest | North Central | SMR | No-take | Non-targeted | 0.357 | 0.03 | *<0.001* | 0.297 | 0.416 | 3 | 0.002 | 12.687 |
| Kelp forest | Central | SMCA | Partial-take | Targeted | -0.271 | 0.331 | 0.414 | -0.92 | 0.379 | 2 | 0.158 | 3.432 |
| Kelp forest | Central | SMR | No-take | Targeted | 0.433 | 0.218 | *0.048* | 0.005 | 0.861 | 10 | 0.356 | 55.411 |
| Kelp forest | Central | SMCA | Partial-take | Non-targeted | -0.087 | 0.447 | 0.845 | -0.964 | 0.79 | 2 | 0.352 | 8.177 |
| Kelp forest | Central | SMR | No-take | Non-targeted | 0.202 | 0.121 | 0.094 | -0.034 | 0.439 | 10 | 0.092 | 66.286 |
| Kelp forest | South | SMCA | Partial-take | Targeted | 0.376 | 0.511 | 0.462 | -0.625 | 1.378 | 5 | 1.149 | 41.689 |
| Kelp forest | South | SMR | No-take | Targeted | 0.571 | 0.246 | *0.02* | 0.089 | 1.053 | 20 | 0.993 | 452.604 |
| Kelp forest | South | SMCA | Partial-take | Non-targeted | -0.291 | 0.534 | 0.586 | -1.338 | 0.757 | 5 | 1.289 | 28.022 |
| Kelp forest | South | SMR | No-take | Non-targeted | 0.062 | 0.225 | 0.784 | -0.38 | 0.504 | 20 | 0.859 | 203.169 |
| Shallow reef | North | SMR | No-take | Targeted | 0.598 | 0.149 | *<0.001* | 0.305 | 0.891 | 2 | 0 | 0.034 |
| Shallow reef | North Central | SMR | No-take | Targeted | 0.841 | 0.313 | *0.007* | 0.229 | 1.454 | 3 | 0.251 | 26.717 |
| Shallow reef | Central | SMR | No-take | Targeted | 0.891 | 0.287 | *0.002* | 0.329 | 1.454 | 4 | 0.3 | 38.546 |
| Shallow reef | South | SMR | No-take | Targeted | 0.858 | 0.299 | *0.004* | 0.271 | 1.444 | 6 | 0.503 | 62.858 |
| Deep reef | North | SMCA | Partial-take | Targeted | 1.359 | 0.392 | *<0.001* | 0.592 | 2.127 | 1 | 0 | 0 |
| Deep reef | North | SMR | No-take | Targeted | -0.152 | 0.334 | 0.648 | -0.807 | 0.502 | 2 | 0 | 0.233 |
| Deep reef | North | SMCA | Partial-take | Non-targeted | 1.355 | 0.003 | *<0.001* | 1.349 | 1.362 | 1 | 0 | 0 |
| Deep reef | North | SMR | No-take | Non-targeted | -0.412 | 0.204 | 0.043 | -0.812 | -0.013 | 2 | 0.053 | 1.833 |
| Deep reef | North Central | SMCA | Partial-take | Targeted | 0.524 | 0.283 | 0.064 | -0.031 | 1.079 | 1 | 0 | 0 |
| Deep reef | North Central | SMR | No-take | Targeted | -0.133 | 0.186 | 0.477 | -0.498 | 0.233 | 2 | 0 | 0.989 |
| Deep reef | North Central | SMCA | Partial-take | Non-targeted | 0.537 | 0.049 | *<0.001* | 0.442 | 0.632 | 1 | 0 | 0 |
| Deep reef | North Central | SMR | No-take | Non-targeted | 0.478 | 0.033 | *<0.001* | 0.414 | 0.542 | 1 | 0 | 0 |
| Deep reef | Central | SMR | No-take | Targeted | 0.083 | 0.344 | 0.809 | -0.592 | 0.758 | 7 | 0.633 | 26.229 |
| Deep reef | Central | SMR | No-take | Non-targeted | 0.306 | 0.134 | *0.023* | 0.043 | 0.568 | 7 | 0.093 | 105.387 |
| Deep reef | South | SMR | No-take | Targeted | 0.484 | 0.23 | *0.035* | 0.035 | 0.934 | 6 | 0.197 | 14.132 |
| Deep reef | South | SMR | No-take | Non-targeted | 0.506 | 0.179 | *0.005* | 0.154 | 0.857 | 6 | 0.108 | 17.21 |

**Appendix S13.** MPA-level meta-analysis results from meta-analyses pooled across MPAs within an ecosystem, region, and target status. The p-values (p) in red text indicates statistically significant relationships.

|  | **Region** | **MPA name** | **Target status** | **Effect size** | **Standard error** | **P-value** | **95% lower** | **95% upper** | **Tau-2** | **Q** | **Ecosystem (latest year)** |
| --- | --- | --- | --- | --- | --- | --- | --- | --- | --- | --- | --- |
| 1 | North | Point Cabrillo SMR | Targeted | -0.374 | 0.35 | 0.285 | -1.06 | 0.311 | 0 | 0 | Kelp forest (2019) |
| 1 | North | Point Cabrillo SMR | Non-targeted | 0.323 | 0.029 | *<0.001* | 0.266 | 0.38 | 0 | 0 | Kelp forest (2019) |
| 2 | North | Sea Lion Gulch SMR | Targeted | -0.296 | 0.447 | 0.508 | -1.173 | 0.581 | 0 | 0 | Deep reef (2014) |
| 2 | North | Sea Lion Gulch SMR | Non-targeted | -0.786 | 0.358 | *0.028* | -1.487 | -0.085 | 0 | 0 | Deep reef (2014) |
| 3 | North | Ten Mile SMR | Targeted | 0.349 | 0.235 | 0.137 | -0.111 | 0.809 | 0.139 | 7.352 | Surf zone (2019), Kelp forest (2020), Shallow reef (2020), Deep reef (2014) |
| 3 | North | Ten Mile SMR | Non-targeted | 0.252 | 0.311 | 0.419 | -0.358 | 0.862 | 0.281 | 488.561 | Surf zone (2020), Kelp forest (2020), Deep reef (2014) |
| 4 | North | Reading Rock SMCA | Targeted | 0.511 | 0.698 | 0.463 | -0.856 | 1.879 | 0 | 0 | Surf zone (2020) |
| 4 | North | Reading Rock SMCA | Non-targeted | 2.903 | 0.235 | *<0.001* | 2.442 | 3.365 | 0 | 0 | Surf zone (2020) |
| 5 | North | South Cape Mendocino SMR | Targeted | 0.558 | 0.26 | *0.032* | 0.049 | 1.068 | 0 | 0 | Shallow reef (2020) |
| 6 | North | Pyramid Point SMCA | Targeted | 1.199 | 0.22 | *<0.001* | 0.768 | 1.631 | 0 | 0 | Kelp forest (2018) |
| 6 | North | Pyramid Point SMCA | Non-targeted | -0.731 | 0.788 | 0.354 | -2.275 | 0.813 | 0 | 0 | Kelp forest (2018) |
| 7 | North | Point St. George Reef Offshore SMCA | Targeted | 1.359 | 0.392 | *0.001* | 0.592 | 2.127 | 0 | 0 | Deep reef (2014) |
| 7 | North | Point St. George Reef Offshore SMCA | Non-targeted | 1.355 | 0.003 | *<0.001* | 1.349 | 1.362 | 0 | 0 | Deep reef (2014) |
| 8 | North | Samoa SMCA | Targeted | 4.988 | 0.922 | *<0.001* | 3.181 | 6.795 | 0 | 0 | Surf zone (2020) |
| 8 | North | Samoa SMCA | Non-targeted | 0.854 | 0.592 | 0.149 | -0.307 | 2.015 | 0 | 0 | Surf zone (2020) |
| 9 | North Central | Salt Point SMCA | Targeted | -0.476 | 0.317 | 0.134 | -1.098 | 0.146 | 0 | 0 | Kelp forest (2011) |
| 9 | North Central | Salt Point SMCA | Non-targeted | 0.25 | 0.022 | *<0.001* | 0.207 | 0.294 | 0 | 0 | Kelp forest (2011) |
| 10 | North Central | Saunders Reef SMCA | Targeted | 0.026 | 0.848 | 0.976 | -1.637 | 1.689 | 0 | 0 | Kelp forest (2020) |
| 10 | North Central | Saunders Reef SMCA | Non-targeted | -0.719 | 0.039 | *<0.001* | -0.797 | -0.642 | 0 | 0 | Kelp forest (2020) |
| 11 | North Central | Bodega Head SMR | Targeted | 0.222 | 0.484 | 0.647 | -0.727 | 1.17 | 0.395 | 6.25 | Shallow reef (2020), Deep reef (2019) |
| 11 | North Central | Bodega Head SMR | Non-targeted | 0.478 | 0.033 | *<0.001* | 0.414 | 0.542 | 0 | 0 | Deep reef (2019) |
| 12 | North Central | Southeast Farallon Island SMR | Targeted | 0.35 | 0.153 | *0.022* | 0.05 | 0.65 | 0 | 0.141 | Shallow reef (2018), Deep reef (2011) |
| 13 | North Central | Del Mar Landing SMR | Targeted | 0.38 | 0.388 | 0.327 | -0.38 | 1.141 | 0 | 0 | Kelp forest (2011) |
| 13 | North Central | Del Mar Landing SMR | Non-targeted | 0.354 | 0.028 | *<0.001* | 0.3 | 0.408 | 0 | 0 | Kelp forest (2011) |
| 14 | North Central | Pillar Point SMCA | Targeted | 0.524 | 0.283 | 0.064 | -0.031 | 1.079 | 0 | 0 | Deep reef (2015) |
| 14 | North Central | Pillar Point SMCA | Non-targeted | 0.537 | 0.049 | *<0.001* | 0.442 | 0.632 | 0 | 0 | Deep reef (2015) |
| 15 | North Central | Stewarts Point SMR | Targeted | 0.55 | 0.825 | 0.505 | -1.066 | 2.166 | 1.317 | 31.131 | Kelp forest (2019), Shallow reef (2020) |
| 15 | North Central | Stewarts Point SMR | Non-targeted | 0.3 | 0.029 | *<0.001* | 0.244 | 0.357 | 0 | 0 | Kelp forest (2019) |
| 16 | North Central | Sea Lion Cove SMCA | Targeted | 0.875 | 1.059 | 0.409 | -1.2 | 2.951 | 0 | 0 | Kelp forest (2011) |
| 17 | North Central | Point Reyes SMR | Targeted | 1.053 | 0.454 | *0.02* | 0.162 | 1.944 | 0 | 0 | Surf zone (2020) |
| 17 | North Central | Point Reyes SMR | Non-targeted | -0.525 | 0.216 | *0.015* | -0.949 | -0.101 | 0 | 0 | Surf zone (2020) |
| 18 | North Central | Point Arena SMR | Targeted | 2.192 | 0.582 | *<0.001* | 1.051 | 3.334 | 0 | 0 | Kelp forest (2011) |
| 18 | North Central | Point Arena SMR | Non-targeted | 0.402 | 0.01 | *<0.001* | 0.382 | 0.422 | 0 | 0 | Kelp forest (2011) |
| 19 | Central | Carmel Bay SMCA | Targeted | -0.562 | 0.19 | *0.003* | -0.935 | -0.189 | 0 | 0 | Kelp forest (2020) |
| 19 | Central | Carmel Bay SMCA | Non-targeted | -0.536 | 0.224 | *0.017* | -0.976 | -0.097 | 0 | 0 | Kelp forest (2020) |
| 20 | Central | White Rock SMCA | Targeted | -0.281 | 0.513 | 0.584 | -1.285 | 0.724 | 0 | 0 | Kelp forest (2011) |
| 20 | Central | White Rock SMCA | Non-targeted | 1.102 | 0.523 | *0.035* | 0.077 | 2.127 | 0 | 0 | Kelp forest (2011) |
| 21 | Central | Natural Bridges SMR | Targeted | -0.128 | 0.813 | 0.875 | -1.721 | 1.465 | 1.103 | 6.011 | Surf zone (2020), Kelp forest (2011) |
| 21 | Central | Natural Bridges SMR | Non-targeted | 0.513 | 0.042 | *<0.001* | 0.43 | 0.596 | 0 | 0.015 | Surf zone (2020), Kelp forest (2011) |
| 22 | Central | Point Lobos SMR | Targeted | -0.042 | 0.529 | 0.937 | -1.08 | 0.996 | 0.957 | 69.631 | Surf zone (2020), Kelp forest (2020), Shallow reef (2020), Deep reef (2019) |
| 22 | Central | Point Lobos SMR | Non-targeted | -0.444 | 0.895 | 0.62 | -2.198 | 1.31 | 2.289 | 39.531 | Surf zone (2020), Kelp forest (2020), Deep reef (2019) |
| 23 | Central | Asilomar SMR | Targeted | 0.042 | 0.266 | 0.875 | -0.48 | 0.564 | 0 | 0.482 | Surf zone (2020), Kelp forest (2011), Deep reef (2008) |
| 23 | Central | Asilomar SMR | Non-targeted | 0.282 | 0.287 | 0.325 | -0.28 | 0.845 | 0.218 | 18.753 | Surf zone (2019), Kelp forest (2011), Deep reef (2008) |
| 24 | Central | Cambria SMCA | Targeted | 0.107 | 0.307 | 0.728 | -0.494 | 0.708 | 0 | 0 | Kelp forest (2008) |
| 24 | Central | Cambria SMCA | Non-targeted | 0.359 | 0.218 | 0.1 | -0.069 | 0.786 | 0 | 0 | Kelp forest (2008) |
| 25 | Central | A√±o Nuevo SMR | Targeted | 0.154 | 0.124 | 0.214 | -0.089 | 0.397 | 0 | 1.741 | Surf zone (2020), Shallow reef (2020), Deep reef (2019) |
| 25 | Central | A√±o Nuevo SMR | Non-targeted | 0.497 | 0.051 | *<0.001* | 0.397 | 0.597 | 0 | 0 | Surf zone (2020), Deep reef (2019) |
| 26 | Central | Big Creek SMR | Targeted | 0.206 | 0.839 | 0.806 | -1.439 | 1.851 | 1.23 | 7.348 | Kelp forest (2015), Deep reef (2016) |
| 26 | Central | Big Creek SMR | Non-targeted | 0.291 | 0.297 | 0.326 | -0.29 | 0.873 | 0.018 | 1.055 | Kelp forest (2015), Deep reef (2016) |
| 27 | Central | Piedras Blancas SMR | Targeted | 0.298 | 0.46 | 0.518 | -0.605 | 1.2 | 0.389 | 12.305 | Kelp forest (2008), Shallow reef (2020) |
| 27 | Central | Piedras Blancas SMR | Non-targeted | -0.26 | 0.107 | *0.015* | -0.469 | -0.052 | 0 | 0 | Kelp forest (2008) |
| 28 | Central | Point Sur SMCA | Targeted | 0.493 | 0.609 | 0.418 | -0.701 | 1.687 | 0 | 0 | Deep reef (2008) |
| 28 | Central | Point Sur SMCA | Non-targeted | -0.161 | 0.055 | *0.004* | -0.27 | -0.052 | 0 | 0 | Deep reef (2008) |
| 29 | Central | Point Buchon SMR | Targeted | 0.79 | 0.454 | 0.082 | -0.099 | 1.68 | 0.535 | 14.299 | Kelp forest (2020), Shallow reef (2020), Deep reef (2019) |
| 29 | Central | Point Buchon SMR | Non-targeted | 0.285 | 0.427 | 0.504 | -0.551 | 1.122 | 0.242 | 2.088 | Kelp forest (2020), Deep reef (2019) |
| 30 | Central | Point Sur SMR | Targeted | 0.814 | 0.343 | *0.018* | 0.141 | 1.487 | 0 | 0 | Kelp forest (2020) |
| 30 | Central | Point Sur SMR | Non-targeted | 0.403 | 0.257 | 0.117 | -0.101 | 0.907 | 0 | 0 | Kelp forest (2020) |
| 31 | Central | Lovers Point - Julia Platt SMR | Targeted | 0.887 | 0.224 | *<0.001* | 0.448 | 1.327 | 0 | 0 | Kelp forest (2020) |
| 31 | Central | Lovers Point - Julia Platt SMR | Non-targeted | 0.216 | 0.099 | *0.029* | 0.022 | 0.41 | 0 | 0 | Kelp forest (2020) |
| 32 | Central | Vandenberg SMR | Targeted | 1.202 | 0.512 | *0.019* | 0.198 | 2.206 | 0 | 0 | Kelp forest (2008) |
| 32 | Central | Vandenberg SMR | Non-targeted | -0.116 | 0.165 | 0.483 | -0.44 | 0.208 | 0 | 0 | Kelp forest (2008) |
| 33 | Central | Portuguese Ledge SMCA | Targeted | 2.034 | 0.499 | *<0.001* | 1.057 | 3.011 | 0 | 0 | Deep reef (2019) |
| 33 | Central | Portuguese Ledge SMCA | Non-targeted | 0.554 | 0.083 | *<0.001* | 0.391 | 0.717 | 0 | 0 | Deep reef (2019) |
| 34 | South | Crystal Cove SMCA | Targeted | -1.566 | 0.565 | *0.006* | -2.672 | -0.459 | 0 | 0 | Kelp forest (2020) |
| 34 | South | Crystal Cove SMCA | Non-targeted | -2.631 | 0.659 | *<0.001* | -3.922 | -1.339 | 0 | 0 | Kelp forest (2020) |
| 35 | South | Carrington Point SMR | Targeted | -0.449 | 0.68 | 0.509 | -1.782 | 0.884 | 1.313 | 48.876 | Kelp forest (2008), Shallow reef (2020), Deep reef (2019) |
| 35 | South | Carrington Point SMR | Non-targeted | -0.197 | 0.065 | *0.002* | -0.325 | -0.07 | 0 | 0.026 | Kelp forest (2008), Deep reef (2019) |
| 36 | South | Scorpion SMR | Targeted | -0.449 | 0.368 | 0.223 | -1.171 | 0.273 | 0 | 0 | Kelp forest (2020) |
| 36 | South | Scorpion SMR | Non-targeted | -0.408 | 0.143 | *0.004* | -0.688 | -0.128 | 0 | 0 | Kelp forest (2020) |
| 37 | South | Blue Cavern Onshore SMCA (No-Take) | Targeted | -0.331 | 0.248 | 0.183 | -0.817 | 0.156 | 0 | 0 | Kelp forest (2020) |
| 37 | South | Blue Cavern Onshore SMCA (No-Take) | Non-targeted | -1.827 | 0.217 | *<0.001* | -2.252 | -1.403 | 0 | 0 | Kelp forest (2020) |
| 38 | South | Cat Harbor SMCA | Targeted | -0.138 | 0.183 | 0.451 | -0.497 | 0.221 | 0 | 0 | Kelp forest (2020) |
| 38 | South | Cat Harbor SMCA | Non-targeted | 0.258 | 0.139 | 0.063 | -0.014 | 0.529 | 0 | 0 | Kelp forest (2020) |
| 39 | South | Point Dume SMCA | Targeted | -0.125 | 0.339 | 0.713 | -0.789 | 0.54 | 0 | 0 | Kelp forest (2020) |
| 39 | South | Point Dume SMCA | Non-targeted | -1.442 | 0.318 | *<0.001* | -2.066 | -0.818 | 0 | 0 | Kelp forest (2020) |
| 40 | South | Begg Rock SMR | Targeted | 0.094 | 0.719 | 0.896 | -1.315 | 1.504 | 0 | 0 | Kelp forest (2013) |
| 40 | South | Begg Rock SMR | Non-targeted | -0.866 | 0.848 | 0.308 | -2.528 | 0.797 | 0 | 0 | Kelp forest (2013) |
| 41 | South | Campus Point SMCA (No-Take) | Targeted | 0.289 | 0.228 | 0.206 | -0.158 | 0.737 | 0 | 0.02 | Surf zone (2020), Kelp forest (2020), Deep reef (2019) |
| 41 | South | Campus Point SMCA (No-Take) | Non-targeted | 0.407 | 0.046 | *<0.001* | 0.316 | 0.498 | 0 | 0.872 | Surf zone (2020), Kelp forest (2020), Deep reef (2019) |
| 42 | South | Matlahuayl SMR | Targeted | 0.511 | 0.767 | 0.505 | -0.992 | 2.014 | 0.938 | 4.936 | Surf zone (2020), Kelp forest (2020) |
| 42 | South | Matlahuayl SMR | Non-targeted | -0.115 | 0.225 | 0.609 | -0.557 | 0.326 | 0.041 | 1.62 | Surf zone (2020), Kelp forest (2020) |
| 43 | South | Farnsworth Onshore SMCA | Targeted | 0.516 | 0.383 | 0.179 | -0.236 | 1.267 | 0 | 0 | Kelp forest (2019) |
| 43 | South | Farnsworth Onshore SMCA | Non-targeted | 0.159 | 0.287 | 0.578 | -0.402 | 0.721 | 0 | 0 | Kelp forest (2019) |
| 44 | South | Santa Barbara Island SMR | Targeted | 0.558 | 0.26 | *0.032* | 0.048 | 1.069 | 0 | 0 | Kelp forest (2019) |
| 44 | South | Santa Barbara Island SMR | Non-targeted | -0.947 | 0.543 | 0.081 | -2.012 | 0.118 | 0 | 0 | Kelp forest (2019) |
| 45 | South | Harris Point SMR | Targeted | 0.647 | 0.262 | *0.013* | 0.134 | 1.16 | 0 | 0.412 | Kelp forest (2020), Deep reef (2019) |
| 45 | South | Harris Point SMR | Non-targeted | 0.827 | 0.121 | *<0.001* | 0.59 | 1.065 | 0 | 0.487 | Kelp forest (2020), Deep reef (2019) |
| 46 | South | South La Jolla SMR | Targeted | 0.689 | 0.15 | *<0.001* | 0.395 | 0.982 | 0 | 0.103 | Kelp forest (2020), Shallow reef (2020) |
| 46 | South | South La Jolla SMR | Non-targeted | 2.949 | 1.059 | *0.005* | 0.874 | 5.025 | 0 | 0 | Kelp forest (2020) |
| 47 | South | South Point SMR | Targeted | 0.703 | 0.203 | *0.001* | 0.305 | 1.101 | 0 | 0.665 | Kelp forest (2020), Deep reef (2019) |
| 47 | South | South Point SMR | Non-targeted | 0.599 | 0.342 | 0.08 | -0.071 | 1.269 | 0.173 | 3.787 | Kelp forest (2020), Deep reef (2019) |
| 48 | South | Anacapa Island SMR | Targeted | 0.726 | 1.082 | 0.502 | -1.394 | 2.846 | 2.276 | 36.57 | Kelp forest (2020), Shallow reef (2020) |
| 48 | South | Anacapa Island SMR | Non-targeted | 0.187 | 0.162 | 0.248 | -0.13 | 0.505 | 0 | 0 | Kelp forest (2020) |
| 49 | South | Abalone Cove SMCA | Targeted | 0.743 | 0.701 | 0.289 | -0.631 | 2.117 | 0 | 0 | Kelp forest (2020) |
| 49 | South | Abalone Cove SMCA | Non-targeted | 0.038 | 0.384 | 0.921 | -0.714 | 0.79 | 0 | 0 | Kelp forest (2020) |
| 50 | South | Point Conception SMR | Targeted | 0.748 | 0.623 | 0.23 | -0.473 | 1.97 | 1.409 | 53.747 | Surf zone (2020), Kelp forest (2012), Shallow reef (2018), Deep reef (2019) |
| 50 | South | Point Conception SMR | Non-targeted | -0.277 | 0.177 | 0.117 | -0.623 | 0.07 | 0 | 0.156 | Surf zone (2020), Kelp forest (2012), Deep reef (2019) |
| 51 | South | Long Point SMR | Targeted | 0.803 | 0.671 | 0.231 | -0.511 | 2.118 | 0 | 0 | Kelp forest (2020) |
| 51 | South | Long Point SMR | Non-targeted | -0.553 | 0.286 | 0.053 | -1.113 | 0.008 | 0 | 0 | Kelp forest (2020) |
| 52 | South | Anacapa Island SMCA | Targeted | 0.895 | 0.51 | 0.079 | -0.105 | 1.894 | 0 | 0 | Kelp forest (2009) |
| 52 | South | Anacapa Island SMCA | Non-targeted | -0.136 | 0.205 | 0.509 | -0.538 | 0.267 | 0 | 0 | Kelp forest (2009) |
| 53 | South | Gull Island SMR | Targeted | 1.087 | 0.316 | *0.001* | 0.467 | 1.706 | 0 | 0.174 | Kelp forest (2020), Deep reef (2019) |
| 53 | South | Gull Island SMR | Non-targeted | 0.472 | 0.194 | *0.015* | 0.092 | 0.853 | 0 | 0.026 | Kelp forest (2020), Deep reef (2019) |
| 54 | South | Laguna Beach SMR | Targeted | 1.147 | 0.173 | *<0.001* | 0.807 | 1.487 | 0 | 0.616 | Surf zone (2020), Shallow reef (2017) |
| 54 | South | Laguna Beach SMR | Non-targeted | -0.087 | 0.317 | 0.785 | -0.708 | 0.535 | 0 | 0 | Surf zone (2020) |
| 55 | South | Naples SMCA | Targeted | 1.17 | 0.409 | *0.004* | 0.367 | 1.972 | 0 | 0 | Kelp forest (2020) |
| 55 | South | Naples SMCA | Non-targeted | 1.053 | 0.506 | *0.037* | 0.061 | 2.045 | 0 | 0 | Kelp forest (2020) |
| 56 | South | Painted Cave SMCA | Targeted | 1.322 | 0.227 | *<0.001* | 0.878 | 1.766 | 0 | 0 | Kelp forest (2020) |
| 56 | South | Painted Cave SMCA | Non-targeted | -0.354 | 0.178 | *0.047* | -0.704 | -0.005 | 0 | 0 | Kelp forest (2020) |
| 57 | South | Point Dume SMR | Targeted | 1.556 | 0.786 | *0.048* | 0.016 | 3.096 | 0.999 | 5.181 | Surf zone (2020), Kelp forest (2020) |
| 57 | South | Point Dume SMR | Non-targeted | 0.978 | 0.3 | *0.001* | 0.39 | 1.566 | 0 | 0.318 | Surf zone (2020), Kelp forest (2020) |
| 58 | South | Point Vicente SMCA (No-Take) | Targeted | 1.561 | 0.412 | *<0.001* | 0.753 | 2.369 | 0 | 0 | Kelp forest (2020) |
| 58 | South | Point Vicente SMCA (No-Take) | Non-targeted | 1.619 | 0.239 | *<0.001* | 1.152 | 2.087 | 0 | 0 | Kelp forest (2020) |
| 59 | South | Swami's SMCA | Targeted | 2.025 | 0.836 | *0.015* | 0.385 | 3.664 | 1.378 | 64.912 | Kelp forest (2019), Shallow reef (2020) |
| 59 | South | Swami's SMCA | Non-targeted | 2.201 | 0.579 | *<0.001* | 1.065 | 3.336 | 0 | 0 | Kelp forest (2019) |

**Appendix S14.** Results from a meta-generalized additive model exploring features of MPA performance.

###
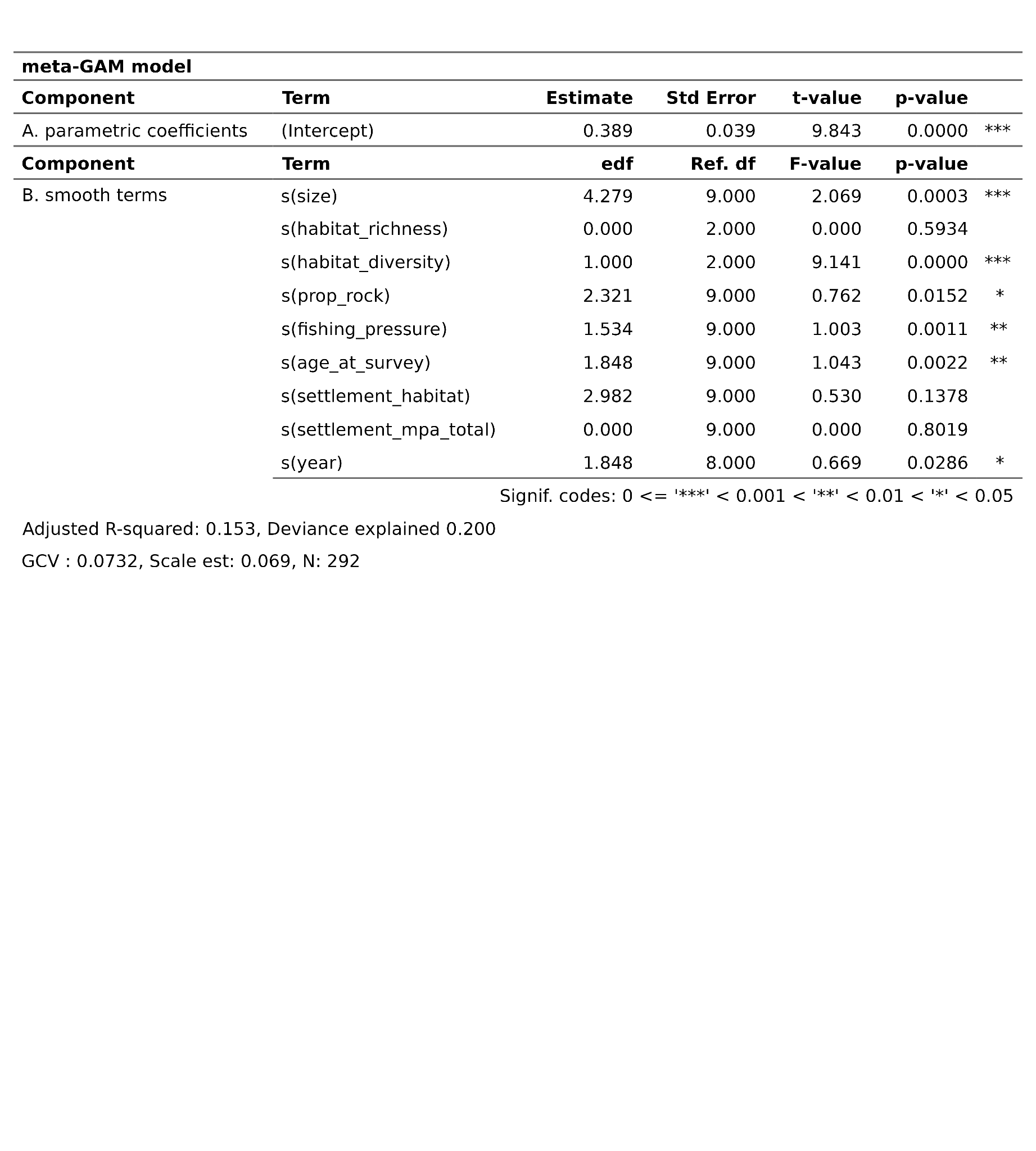


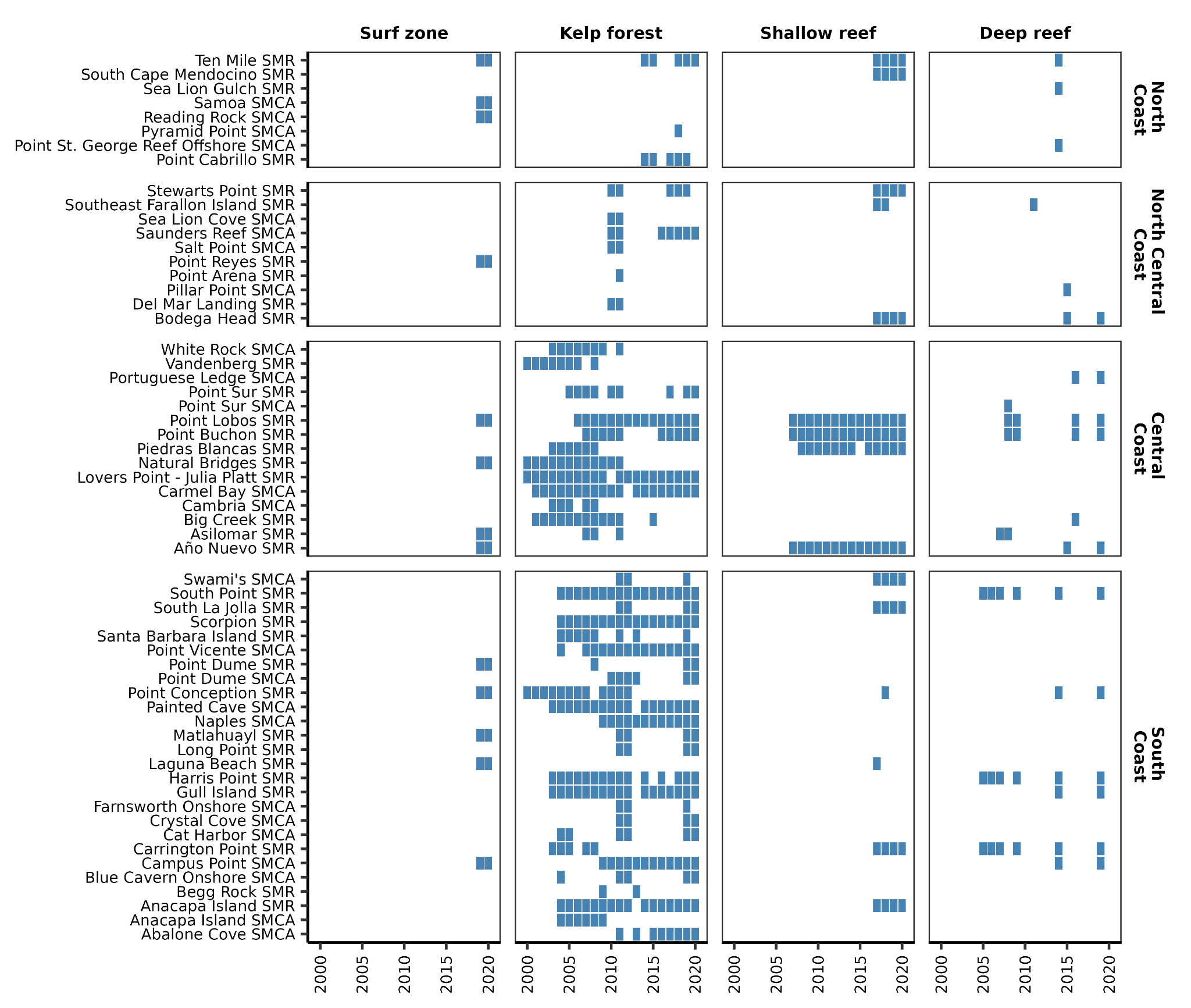


**Appendix S15.** Distribution of long-term monitoring effort by marine protected area (MPA), region, and ecosystem. Cell color indicates the number of surveys conducted each year in each MPA and ecosystem with paired reference sites. White cells indicate that no surveys were conducted. SMR = State Marine Reserve (no-take MPA); SMCA = State Marine Conservation Area (partial-take MPA, with a few official and habitat-specific de facto exceptions).


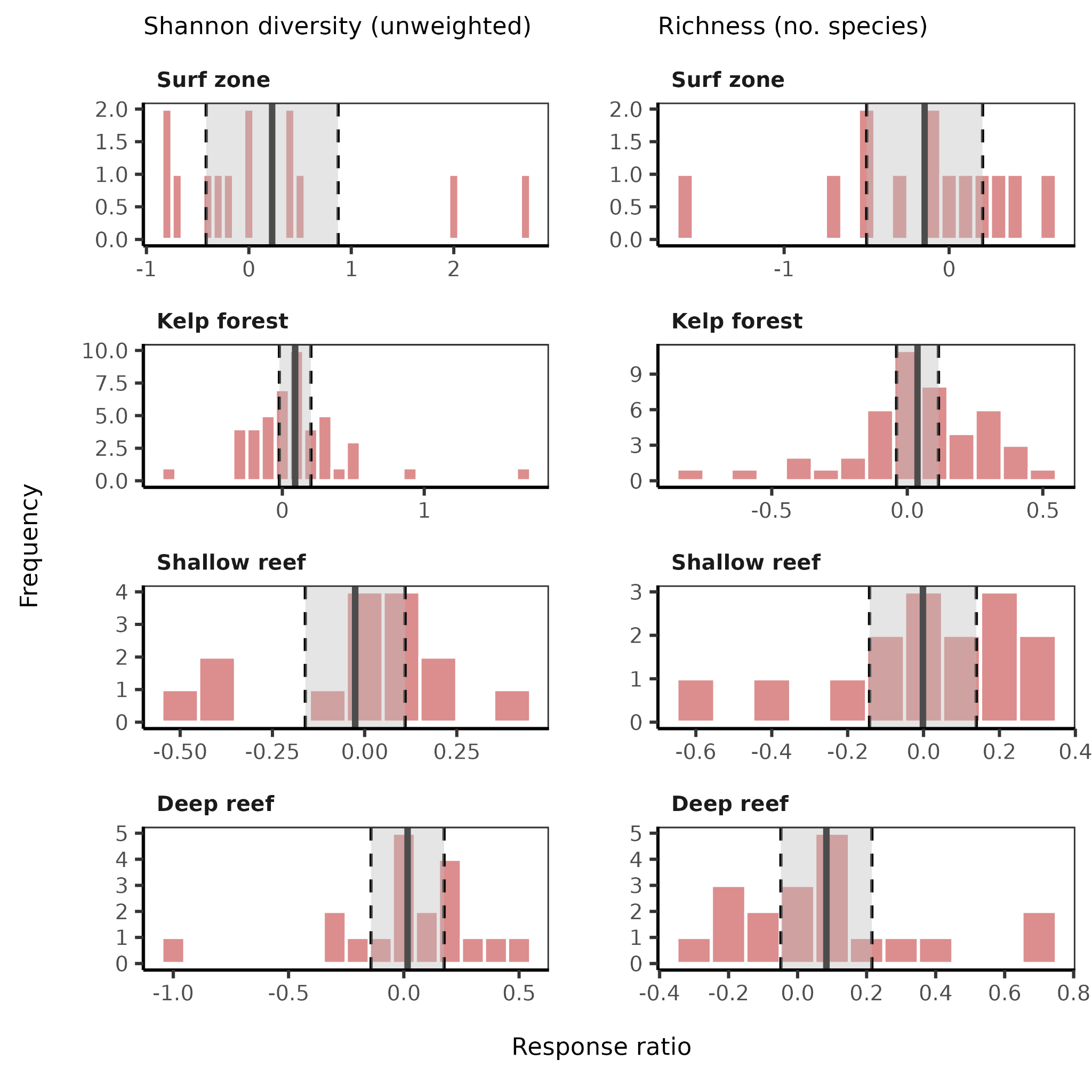


**Appendix S16.** Shannon diversity (left) and richness (right) response ratios for four ecosystems (surf zone, kelp forest, shallow reef, deep reef). Each bar represents the response ratio frequency for the most recent year of sampling for each MPA. Solid vertical lines represent the mean response ratio, and the shaded areas indicate the 95% confidence interval. Confidence regions for all ecosystems overlap with 0, indicating that species diversity and richness are not significantly distinguishable between MPAs and areas that allow fishing.

**
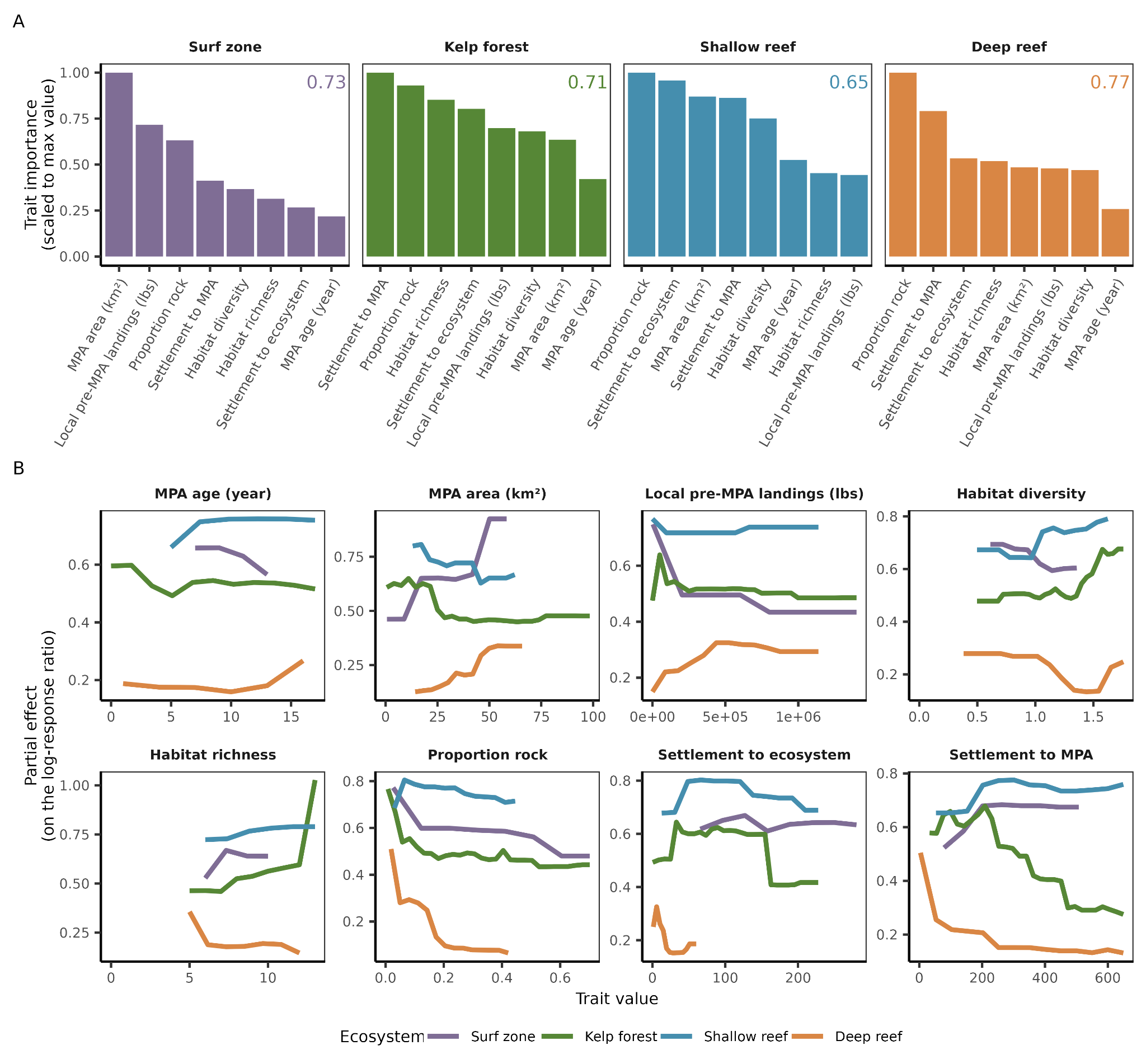
**

**Appendix S17.** The **(A)** importance and **(B)** partial effects of MPA features on surf zone (purple), kelp forest (green), shallow reef (blue), and deep reef (orange) ecosystem-specific conservation performance (log-response ratio) for targeted species estimated by the random forest models. In **(A)**, feature importance is measured as the mean decrease in node impurity resulting from splitting on each feature scaled to the maximum value. MPA features are sorted in order of decreasing importance. The r^2^ of each model fit is printed in the top-right corner of each plot. In **(B)**, lines indicate the ecosystem-specific impact of varying each MPA feature on conservation performance (log-response ratio) holding other features at their means. Panels are arranged in descending rank-order (MPA age = highest, settlement to MPA = lowest) based on the average impurity score across ecosystems.

**References**

Auster, P. J., Malatesta, R. J., & Donaldson, C. L. S. (1997). Distributional responses to small-scale habitat variability by early juvenile silver hake, Merluccius bilinearis. Environmental Biology of Fishes, 50(2), 195–200.

Caselle, J. E., Rassweiler, A., Hamilton, S. L., & Warner, R. R. (2015). Recovery trajectories of kelp forest animals are rapid yet spatially variable across a network of temperate marine protected areas. Scientific Report, 5, 14102.

Hamilton, S. L., Caselle, J. E., Malone, D. P., & Carr, M. H. (2010). Incorporating biogeography into evaluations of the Channel Islands marine reserve network. Proceedings of the National Academy of Sciences, 107(43), 18272– 18277.

Liaw, A., & Wiener, M. (2002). Classification and regression by randomForest. R News, 2(3), 18–22.

Lindholm, J., Auster, P., & Valentine, P. (2004). Role of a large marine protected area for conserving landscape attributes of sand habitats on Georges Bank (NW Atlantic). Marine Ecology Progress Series, 269, 61–68.

Malone, D. P., Davis, K., Lonhart, S. I., Parsons-Field, A., Caselle, J. E., & Carr, M. H. (2022). Large-scale, multidecade monitoring data from kelp forest ecosystems in California and Oregon (USA). Ecology, 103(5), e3630.

Smith, J. G., Free, C. M., Lopazanski, C., Brun, J., Anderson, C. R., Carr, M. H., Claudet, J., Dugan, J. E., Eurich, J. G., Francis, T. B., Hamilton, S. L., Mouillot, D., Raimondi, P. T., Starr, R. M., Ziegler, S. L., Nickols, K. J., & Caselle, J. E. (2023). A marine protected area network does not confer community structure resilience to a marine heatwave across coastal ecosystems. Global Change Biology, 29(19), 5634–5651.

Starr, R. M., Wendt, D. E., Barnes, C. L., Marks, C. I., Malone, D., Waltz, G., Schmidt, K. T., Chiu, J., Launer, A. L., Hall, N. C., & Yochum, N. (2015). Variation in responses of fishes across multiple reserves within a network of marine protected areas in temperate waters. PLoS ONE, 10(3), e0118502.

Yochum, N., Starr, R. M., & Wendt, D. E. (2011). Utilizing fishermen knowledge and expertise: Keys to success for collaborative fisheries research. Fisheries, 36(12), 593–605.

Ziegler, S. L., Brooks, R. O., Hamilton, S. L., Ruttenberg, B. I., Chiu, J. A., Fields, R. T., Waltz, G. T., Shen, C., Wendt, D. E., & Starr, R. M. (2022). External fishing effort regulates positive effects of no-take marine protected areas. Biological Conservation, 269, 109546.
